# A Variant of *GJD2*, Encoding for Connexin 36, Alters the Function of Insulin Producing β-Cells

**DOI:** 10.1371/journal.pone.0150880

**Published:** 2016-03-09

**Authors:** Valentina Cigliola, Celine Populaire, Ciro L. Pierri, Samuel Deutsch, Jacques-Antoine Haefliger, João Fadista, Valeriya Lyssenko, Leif Groop, Rico Rueedi, Fabrizio Thorel, Pedro Luis Herrera, Paolo Meda

**Affiliations:** 1 Department of Genetic Medicine and Development, University of Geneva Faculty of Medicine, Geneva, Switzerland; 2 Centre Hospitalier Régional Universitaire Besançon, Besançon, France; 3 Department of Biosciences, Biotechnologies and Biopharmaceutics, University of Bari, Bari, Italy; 4 Joint Genome Institute, Walnut Creek, California, United States of America; 5 Department of Medicine, CHUV, Lausanne, Switzerland; 6 Department of Clinical Sciences, Diabetes and Endocrinology, Lund University, Malmö, Sweden; 7 Steno Diabetes Center A/S, Gentofte, Denmark; 8 Department of Computational Biology, University of Lausanne, Rue du Bugnon 27, 1011, Lausanne, Switzerland; 9 Swiss Institute of Bioinformatics, 1015, Lausanne, Switzerland; 10 Department of Cell Physiology and Metabolism, University of Geneva Faculty of Medicine, Geneva, Switzerland; Universidade Federal do ABC, BRAZIL

## Abstract

Signalling through gap junctions contributes to control insulin secretion and, thus, blood glucose levels. Gap junctions of the insulin-producing β-cells are made of connexin 36 (Cx36), which is encoded by the *GJD2* gene. Cx36-null mice feature alterations mimicking those observed in type 2 diabetes (T2D). *GJD2* is also expressed in neurons, which share a number of common features with pancreatic β-cells. Given that a synonymous exonic single nucleotide polymorphism of human Cx36 (SNP *rs3743123*) associates with altered function of central neurons in a subset of epileptic patients, we investigated whether this SNP also caused alterations of β-cell function. Transfection of *rs3743123* cDNA in connexin-lacking HeLa cells resulted in altered formation of gap junction plaques and cell coupling, as compared to those induced by wild type (WT) *GJD2* cDNA. Transgenic mice expressing the very same cDNAs under an insulin promoter revealed that SNP *rs3743123* expression consistently lead to a post-natal reduction of islet Cx36 levels and β-cell survival, resulting in hyperglycemia in selected lines. These changes were not observed in sex- and age-matched controls expressing WT hCx36. The variant *GJD2* only marginally associated to heterogeneous populations of diabetic patients. The data document that a silent polymorphism of *GJD2* is associated with altered β-cell function, presumably contributing to T2D pathogenesis.

## Introduction

Gap junctional channels are composed of connexin (Cx) proteins, and allow for the communication between adjacent cells through the diffusion of cytosolic ions and small molecules [1, 2]. Cx36 is the main connexin isoform expressed in neurons and pancreatic β cells [3–8], and previous studies have provided evidence that alterations of Cx36 signalling profoundly affects the function and survival of these two cell types [9, 10]. Thus, deletion of Cx36 results in loss of gap junctions between fast-spiking interneurons of hippocampus and cortex, and interferes with their oscillatory activity [11, 12]. This deletion also impairs the coupling of amacrine and bipolar neurons of retina, resulting in vision alterations [13, 14] and increased retinal vulnerability [15]. In pancreatic islets, loss of Cx36 alters the regular Ca^2+^ oscillations which are induced in β cells during glucose stimulation, as well as basal, first and second phases of insulin release [16], resulting in glucose intolerance [17]. The absence of Cx36 also leads to increased apoptosis of β cells after exposure to cytotoxic drugs and cytokines [18, 19], and to decreased transcriptional regulation of the insulin gene [4]. Several of these alterations mimic signs of type 2 diabetes (T2D) [20].

A single-nucleotide polymorphism (SNP *rs3743123)* in the coding sequence of the *GJD2* gene, which encodes for human Cx36 (hCx36), has been associated with juvenile myoclonic epilepsy [21, 22]. Alike type 2 diabetes, this form of epilepsy has a complex inheritance pattern [23].

Given the numerous similarities between neurons and β cells [24], we investigated whether the same *GJD2* polymorphism may alter the function of the insulin-producing β cells, sufficiently to cause an abnormal regulation of glycaemia. By generating novel lines of HeLa cells stably expressing either the WT or the SNP *rs3743123* variant of hCx36, we found that the latter reduced the gap junction-mediated coupling of β cells and altered the packing of Cx36 channels in the cell membrane. By generating novel lines of transgenic mice, we further observed that the variant form of hCx36 decreased after birth, consistently resulting in loss of β cells which, in some lines, lead to sustained hyperglycemia. By investigating a population of Caucasian individuals, we show that the variant *GJD2* is marginally associated to type 2 diabetics.

## Materials and Methods

### Culture and transfection of HeLa cells

HeLa cells (American Type Culture Collection-LGC Promochem, Teddington, Middlesex, UK) were grown in DMEM without glutamax (Gibco 61965), containing 10% (v/v) fetal calf serum (INVITROGEN 10270098), and a 1% penicillin/streptomycin mix (GIBCO 15140). Cells were cultured in 75 cm^2^ culture flasks, and incubated in a 5% CO_2_ humidified environment at 37 C. Once confluent, cells were trypsinized and seeded in 12-well culture plates (5×10^4^ cells per well). Cells were incubated to reach 70% confluence and then transfected with an empty pcDNA3.1 (3.4 μg/μl vector) or with a pcDNA3.1 construct containing 3.8 μg/μl cDNA encoding for either hCx36 WT or hCx36 SNP *rs3743123*, using Lipofectamine 2000 (GIBCO 11668–030) according to the manufacturer's instructions. One day later, the cells were collected and cultured in a medium containing 750 μg/mL G418 (GIBCO 11811–031), which was changed every 2 days. Surviving cells were passed at one week intervals, and plated under limiting dilution conditions (1 cell/well) in 96 well plates, to select individual clones which, thereafter, were cultured for a 3 year period under the continuous pressure selection provided by G418. Three clones, which were shown by immunofluorescence staining and western blot analysis of total proteins to express similar and stable levels of either the WT or the *rs3743123* form of hCx36, were selected for the experiments.

### Predicted folding of Cx36 mRNAs

The most recent annotated Cx36 mRNA sequence (NM_020660.2) of 1096 bp, covering the complete coding region of Cx36, was downloaded from the NCBI. ClustalW [25] implemented in Jalview [26] was used to generate the sequence alignment of WT Cx36 mRNA (681C) and of two allelic variants: variant 681C>T, previously known as 588C>T, and variant 462C>T, previously known as 369C>T. Visual Gene Developer 1.7 program [27] was used to calculate the mRNA structure by employing the “mRNA structure (slow)” tool.

### Generation of transgenic mice

The experiments were carried out in accordance with the animal protection law of the State of Geneva. All animals were housed and cared for according to the guidelines of the Direction Générale de la Santé du Canton de Genève. This study was reviewed and approved by the Commission cantonale pour les expériences sur les animaux and the Direction Générale de la Santé du Canton de Geneva. Mice were euthanized by rapid cervical dislocation.

cDNAs encoding either hCx36 WT or hCx36 SNP *rs3743123* were subcloned into a plasmid containing a 0.7 kb-long fragment of the rat insulin II promoter, and a 1.6 kb-long sequence containing an intron and the polyA signal of the rabbit β-globin gene. After testing the actual expression of the transgenic proteins by transient transfection of the 2 constructs in the RIN cell line [4, 28], the transgenes were excised from the plasmids and injected into the zygotic pronuclei of B6D2/JIcoF1 hybrid mice (C57BL/6J x DBA/2J), which were implanted into pseudo-pregnant NMRI females [29]. Nine and six independent founders (F_0_) were generated using the hCx36 WT and the hCx36 SNP *rs3743123* construct, respectively. Expression of either hCx36 WT or hCx36 SNP *rs3743123* was detected in three and two lines of mice, respectively. Transgenic female mice positive for the transgenes, and heterozygous for mCx36, were then crossed with Cx36-null male mice [16] to obtain litters whose β cells only expressed either the WT (hereafter referred to as RIP-hCx36WT mice) or the *rs3743123* form of hCx36 (hereafter referred to as RIP-hCx36*rs3743123* mice). Mice were then bred to maintain the transgenes at the heterozygous state in all animals, and to avoid homozygous loss of function. We generated three independent lines of RIP-hCx36WT mice and two independent lines of RIP-hCx36*rs3743123* mice (lines A and B), expressing comparable levels of hCx36. The genotype of littermates was determined by PCR amplification of ear DNA [30]. Experiments were initiated with mice of the F2 generation and repeated with mice of the F6 generation. Both males and females were used for the experiments.

### Immunofluorescence

Cells were attached to polylysine (P7280 Sigma) coated glass slides, and fixed for 10 min in 70% ETOH at -20°C. For insulin, glucagon and Cx36 staining, mouse pancreas were fixed for 90 min in 4% paraformaldehyde (PFA), washed in 0.1 M phosphate-buffered saline (PBS), and transferred in PBS containing 25% sucrose overnight. Cryostat sections of 10-μm thickness were incubated for 20 min in PBS containing 0.2% Triton, washed in PBS, incubated for 30 min in PBS supplemented with 2% BSA, and exposed for 2 h at room temperature to one of the following primary antibodies: rabbit polyclonal antibody anti-Cx36 (1:80, Life Technologies), guinea pig polyclonal anti-insulin (1:400, DAKO), mouse monoclonal anti-glucagon (1:1000, SIGMA), rabbit polyclonal anti-somatostatin (1:200, DAKO). After rinsing, sections were incubated with one of the following secondary antibodies: Alexa Fluor^®^ 488 Dye, Alexa Fluor^®^ TRITC Dye, Alexa Fluor^®^ 647 Dye (Invitrogen), whichever required, and all diluted 1:500. DAPI was added to the secondary antibodies. Cell and section immunolabeling were examined with an Axiophot fluorescence microscope (Zeiss) and a Leica TCS SPE confocal microscope (Leica Microsystems, Bannockburn, IL), respectively.

### Electron Microscopy

Cells were fixed for 60 min in 2.5% glutaraldehyde in 0.1 M phosphate buffer at room temperature, and processed for freeze-fracture, as previously described [31, 32].

### RNA extraction and qRT-PCR

Cells were exposed to either 5 μg/ml actinomycin D (SIGMA A9415) in 100% ethanol or to 100% ethanol alone. RNAs were isolated at different time points using Trizol reagent (15596–026, Invitrogen), as per the manufacturer’s instructions. RNA integrity was assessed on agarose gels, and OD was measured using a BioPhotometer 6131. Retrotranscription was performed with 1 μg RNA of each sample, using random hexamers and the SuperScript III Reverse Transcriptase (Invitrogen). PCR reactions were performed using 2x Power SYBR Green Master Mix (Applied Biosystem), and the following primers: for Cx36, CATAATGGTGTGTACCCCCAGTCT (Fw) and CGGCGTTCTCGCTGCTT (Rev); for RPS9, GGGAACTGCTGACGCTTGAT (Fw) and AGGGCGTTGCCTTCGAA (Rev); for GAPDH, ATGGAAATCCCATCACCATCTT (Fw) and CGCCCCACTTGATTTTGG (Rev); for β-actin, CCAGCTCACCATGGATGATG (Fw) and CCAGCTCACCATGGATGATG (Rev). Each reaction was performed in triplicates. Fold changes were calculated using the GeNorm method [33].

### Western Blot

For preparation of total protein extracts, transfected HeLa cells were sonicated 3 times during 10 sec in a 0.1 M Tris-HCl lysis buffer containing 5% SDS, 5 mM EDTA and a mix of protease inhibitors (Roche Applied Science).

For preparation of membrane proteins, HeLa cells were sonicated in a 0.1 M Tris-HCl lysis buffer, pH 7.4, containing 20 mM EDTA and a mix of protease inhibitors. The homogenates were centrifuged at 4000 g for 10 min at 4°C. Supernatants were collected and centrifuged for 90 min at 260’000 g and 4°C. The membrane pellet was suspended in 0.1 M Tris-HCl buffer, pH 7.4, supplemented with 5% SDS and 5 mM EDTA. Membrane proteins were incubated at 95°C for 10 min and sonicated a second time. Protein content was determined by the DC protein assay reagent kit (Bio-Rad). Proteins were heated at 50°C in a loading buffer, separated by electrophoresis in a 10% polyacrylamide gel, and transferred for 60 min onto immobilon polyvinylidene difluoride membranes (Millipore), at a constant voltage of 100 V. Membranes were washed 4 times 15 min in TBS-Tween20 0.1% and then incubated for 30 min at room temperature in TBS containing 5% milk and 0.1% Tween20 (blocking buffer). Then, membranes were incubated overnight at 4°C with an antibody against Cx36, diluted 1:150 or actin (Sigma), diluted 1:250 in blocking buffer. Membranes were washed and incubated with secondary antibodies for 1 hour at room temperature, and antigen-antibody complexes were detected with ECL. In these experiments, negative and positive controls included extracts of wild-type HeLa and MIN6 cells, respectively. Quantifications were performed using the ChemiDoc^™^hemiDoccat (Biorad, Cressier, Switzerland) and the Quantity One software.

### Dye injection

Three day-old cultures of HeLa cells were transferred onto the heated (37°C) stage of an inverted Zeiss ICM35 microscope, and individual cells were microinjected with either 4% Lucifer Yellow (LY) or 4% ethidium bromide (EB) in 150 mM LiCl buffered with 10 mM Hepes-buffered (pH 7.2), as previously reported [34]. Three independent clones of each type of transfected cell were tested, and data pooled for each cell type. Cell coupling extent was calculated after each injection by scoring the number of cells containing one of the two injected tracers, including the injected cell. Cell coupling incidence was determined by calculating the percentage of injections resulting in the cell-to-cell transfer (i.e. more than one cell stained) of one of the two tracers. From these data, we calculated a coupling index for either the LY or the EB tracers, given by the product of mean coupling extent and coupling incidence, and a total coupling index given by the product of the coupling index of LY and that of EB.

### *In vitro* and *in vivo* data analysis

Data are expressed as means + SE of the indicated number of experiments. Statistical analysis was performed using the Statistical Package for Social Science (SPSS 15.0, SPSS inc.). For normally distributed values, differences between means were assessed by analysis of variance, using the *post hoc* Bonferroni test. Coupling extent data were compared using the median test. Coupling indices were compared using the Chi square test. Statistical analysis of data from transgenic mice was performed using GraphPad Prism 6.00 software (GraphPad, San Diego, CA). Differences were considered significant when p<0.05.

### eQTLs of human pancreatic islets

Islets from cadaver organ donors were provided by the Nordic Islet Transplantation Program (www.nordicislets.org), courtesy of Prof. Olle Korsgren, Uppsala University, Sweden. All procedures were approved by the ethics committees of the Uppsala and Lund Universities, and informed written consent obtained from donors or their relatives. The microarray experiments (Human Gene 1.0 ST whole transcript) were performed on islets isolated from 81 normoglycemic individuals, (aged 56.3 ± 1.3 years, and featuring a BMI 25.7 ± 0.4 kg/m^2^, and a HbA1_c_ 5.5 ± 0.04%), and 47 hyperglycemic patients (aged 60.3 ± 1.2 years, and featuring a BMI 27.8±0.6 kg/m^2^, impaired glucose tolerance, and a HbA1_c_ 6.6 ± 0.1%). RNA products were fragmented and hybridized to the GeneChip Human HG U 133A Array (Affymetrix, Santa Clara, CA, USA) [35]. Statistical analyses of expression data were performed using two-tailed Spearman’s t-test.

### CoLaus study

The CoLaus cohort [36] consists of a random sample of 5435 Caucasian women and men, genotyped using the 500 K Affymetrix chip technology. In this cohort, patients were defined as T2D when featuring a fasting plasma glucose ≥ 7.0 mmol/L or receiving an oral hypoglycaemic or insulin treatment. The study was approved by the Institutional Ethics Committee of the University of Lausanne (Commission d’Ethique de la recherche clinique, Sous-Commission I). All participants received an information letter about the study and signed informed consent.

Out of this cohort, we initially analyzed a group of 299 T2D patients and 500 unrelated normoglycaemic subjects (the clinical characteristics of the 2 groups are given in S1 Table), predicted to provide a 70% power of detecting a SNP associated to T2D, assuming that the relative risk conferred by this SNP was about 1.5, i.e. similar to that of previously reported loci. Each SNP was amplified with 10 ng total genomic DNA, using the primers and probes reported in Mas et al. 2004 [22] and a jump start red taq ready mix (Sigma). Amplicons were analysed by the Pyrosequencing^™^ technology (Biotage, Uppsala, Sweden). All amplification reactions were carried out under identical conditions. 26 individuals were genotyped in duplicate to assess genotyping accuracy. All SNPs gave a genotyping concordance rate of 100%. Allele and genotype frequencies were tested for Hardy-Weinberg equilibrium by a Chi-square test. Differences in allele and genotype distributions between cases and controls were compared using Pearson’s chi square. Odds ratios, with 95% CIs and p values, were determined using a Chi-square test. For all quantitative analyses, data were log transformed when appropriate, and a p value less than 0.05 was considered significant. Statistics were performed using Minitab and SPSS (windows version 14.0). Haploview software version 3.32 [37] was used to determine the pairwise linkage disequilibrium and haplotype structure of *GJD2*. The haplotype reconstruction was carried out using the PHASE software [38]. Power calculations were performed using the Genetic Power Calculator [39], assuming a co-dominant model, 5% prevalence and a genotype relative risk of 1.36.

## Results

### SNP *rs3743123* alters Cx36 packing and function, but not half-life *in vitro*

**Fig 1:**
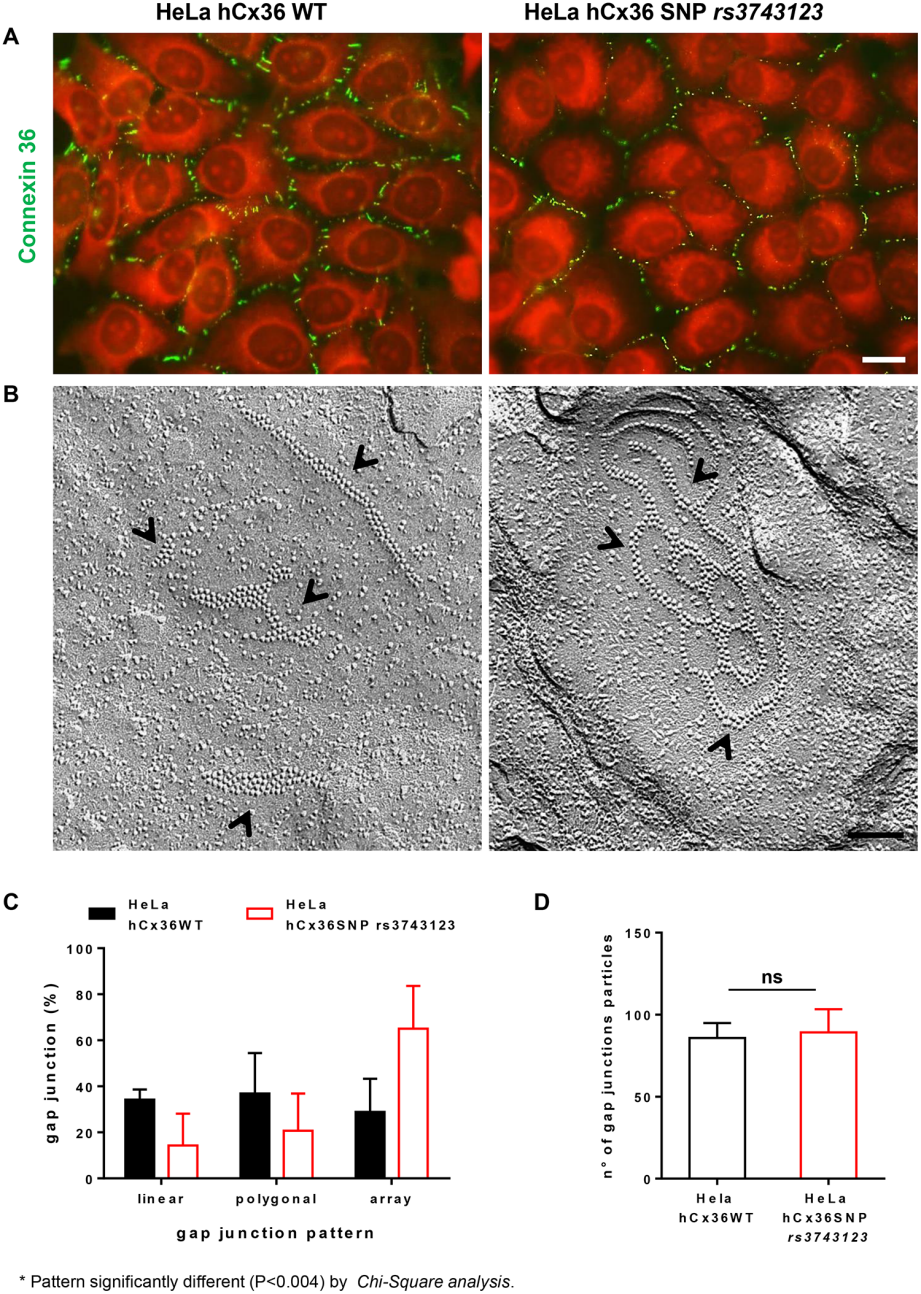
SNP *rs3743123* alters the distribution of Cx36 at the cell membrane. **A**, In HeLa cells stably transfected with the wild type form of Cx36 (left) the protein shows a spotted distribution (green) at the cell membrane. After transfection of the SNP *rs3743123* variant (right) the spotted distribution of the protein alternates with regions of continuous membrane staining. Scale bar, 20 μm. **B**, Freeze-fracture electron microscopy revealed polygonal and array-shaped gap junction plaques (arrow heads) in HeLa cells transfected with either the WT or variant form of Cx36 (right). Scale bar, 85 nm. **C,** Distribution of different gap junction patterns (polygonal, linear, array shaped) and **D,** numbers of particles (connexons) per plaque in HeLa cells transfected with the wild type (n = 37) and SNP *rs3743123* forms of Cx36 (n = 48). Images and mean + SEM data are from three independent clones stably expressing either the wild type or the SNP *rs3743123* form of the protein.

To investigate the effect of SNP *rs3743123*, we transfected HeLa cells, which lack connexin expression ([34], S1 Fig and Table 1), with a cDNA fragment encoding either the WT or the *rs3743123* variant of human *GJD2*. For each of these 2 constructs, we selected 3 stable clones expressing comparable levels of transfected hCx36 (S1 Fig). All clones showed the membrane insertion of the transfected proteins, as assessed by immunoblotting of membrane proteins (S1 Fig), and by immunofluorescence staining of cells (Fig 1A, left panel). The latter approach further revealed that whereas most WT hCx36 featured a spotted distribution at small membrane domains, *rs3743123* hCx36 was often more broadly distributed along the cell membrane (Fig 1A, right panel). Freeze-fracture electron microscopy further revealed polygonal, linear or arrayed gap junction plaques within the membranes of all stably transfected HeLa cell clones (Fig 1B and 1C). While the distribution of these gap junction types differed (p < 0.004) in the cells transfected for WT and variant hCx36, gap junction plaques contained on average a comparable number of connexons in the two cell types (Fig 1D).

**Table 1:** SNP *rs3743123* reduces hCx36 coupling between adjacent cells.

HeLa type	coupling extent ^a^		coupling incidence ^b^ (% injections)		coupling index ^c^		
	LY	EB	LY	EB	LY	EB	total
**wild type**	1.05 ± 0.05 n = 21	1.07 ± 0.05 n = 28	4.8	7.1	8.6	7.6	65.4
**transfected for hCx36** **wild type**	2.00 ± 0.24^§§^ n = 16	3.33 ± 0.32^§§^ n = 42	62.5**	73.8**	125.0	245.7	30’712.5***
**transfected for hCx36** **SNP rs3743123**	1.88 ± 0.15^§^ n = 42	2.38 ± 0.19^§§^ n = 55	59.5**, ^#^	69.1**, ^#^	111.9	164.5	18’407.5***

LY = Lucifer yellow; EB = ethidium bromide. Coupling extent = number of cells labeled by the microinjected tracer (including the injected cell); coupling incidence = percent of injections showing coupling (coupling extent >1); coupling index = mean coupling extent x coupling incidence; total coupling index = coupling index of LY x coupling index of EB.

^a^ Data are mean ± SE of 4 experiments, in which the results from 3 independent and stably-transfected clones were pooled for each cell type; n = number of microinjections.

^§^ p< 0.003,

^§§^ p < 0,001 vs corresponding value in wild type HeLa cells, as evaluated by both ANOVA and median tests.

^b^ values are percent of injections leading to labeling of > 1 cell.

** p < 0.001 vs corresponding value in wild type HeLa cells,

^#^ p < 0.007 vs corresponding value in cells transfected for hCx36 wild type, as evaluated by the non parametric Chi square test.

^c^ ***p < 0.001 vs corresponding value in wild type HeLa cells, as evaluated by the non parametric Chi square test.

Microinjections of ethidium bromide and lucifer yellow, showed a sizable coupling of the transfected cells which, as expected for channels made by Cx36 [34], was larger in all clones when tested with ethidium bromide than with lucifer yellow (Table 1). However, the estimates of coupling extent and incidence were lower in the cells that expressed the variant form of hCx36 than in those which were coupled by WT hCx36 (Table 1).

**Fig 2:**
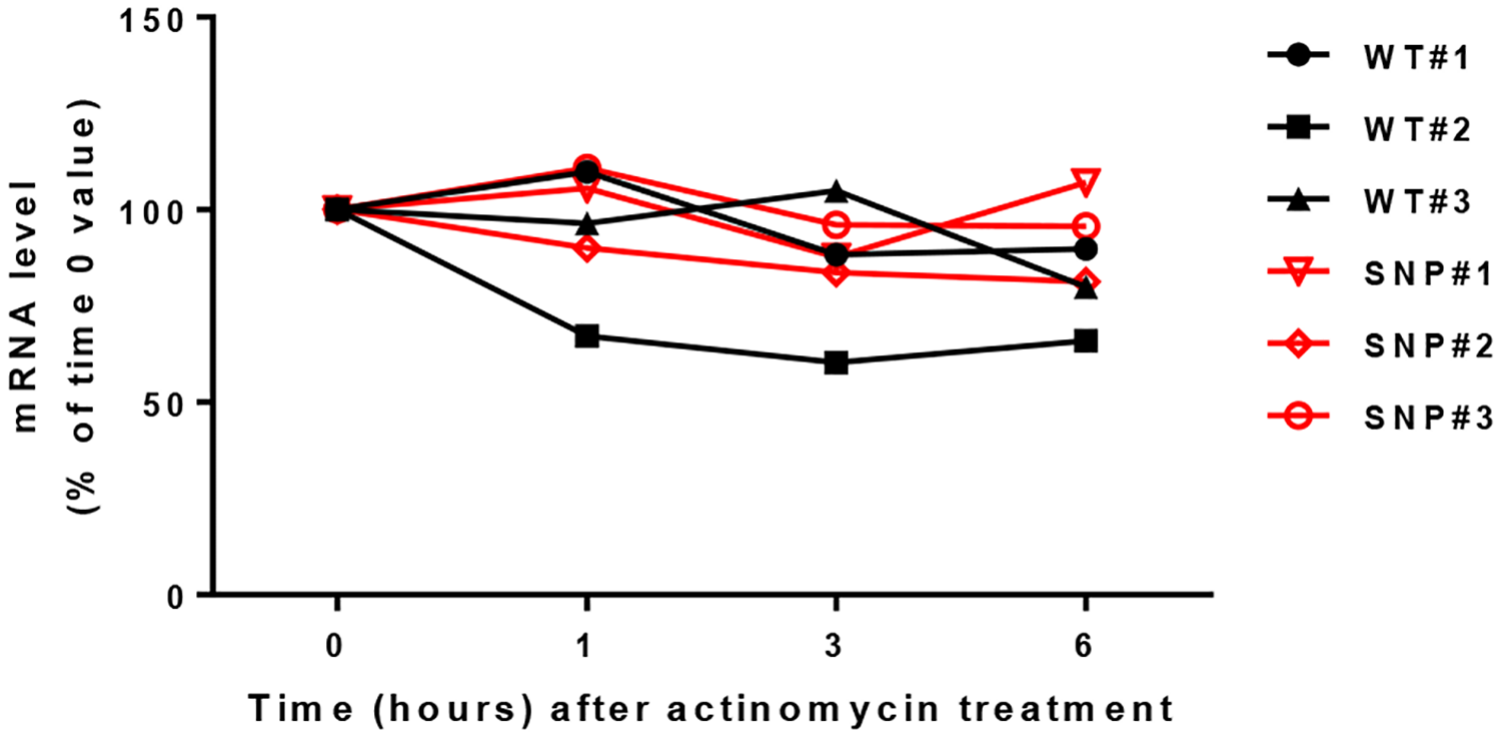
SNP *rs3743123* does not alter the stability of Cx36 mRNA. Exposure to 5 μg/ml actinomycin D, revealed comparable levels of Cx36 mRNA at different time points in 3 independent clones of HeLa cells stably transfected with either the wild type (black symbols) or the SNP *rs3743123* form of hCx36 (red symbols).

Exposure of HeLa cells to the transcription inhibitor actinomycin D, revealed that the transcript levels of both WT and variant hCx36 decayed with a similar time-course (T½ ~about 3 h, Fig 2).

These data show that that SNP *rs3743123* does not interfere with the intracellular trafficking of hCx36, its insertion into the cell membrane and its packaging into functional gap junction plaques. However, it affects gap junction distribution and reduces the extent of intercellular coupling, without altering the native half-life of hCx36 mRNA.

### SNP *rs3743123* induces changes in the predicted folding of Cx36 mRNA

We investigated the most recent annotated version of Cx36 mRNA, where SNP *rs3743123* is attributed to position 681C>T (previously known as 588C>T [22]; S2 Fig). We found that the allelic variant 681T markedly changed the predicted tridimensional structure of the native hCx36 transcript containing a 681C nucleotide (S3 Fig). This conformational change was not observed in the mRNA of hCx36 carrying another SNP *rs35174018* (462 C/T, previously reported as 369 C/T [22]) which, alike SNP *rs3743123*, is also located in exon 2 of hCx36 (S4 Fig). The data show that SNP *rs3743123* specifically alters the predicted structure of human Cx36 mRNA.

### SNP *rs3743123* reduces the post-natal expression of Cx36 and the survival of β cells *in vivo*

To evaluate the effect of SNP *rs3743123 in vivo*, we generated transgenic mice expressing either the WT or the *rs3743123* form of hCx36 specifically in β cells (S5A Fig). These mice were crossed with Cx36-null male mice [16], whose β cells lack endogenous mCx36, to allow for evaluation of the effects solely dependent on either WT or variant hCx36.

**Fig 3:**
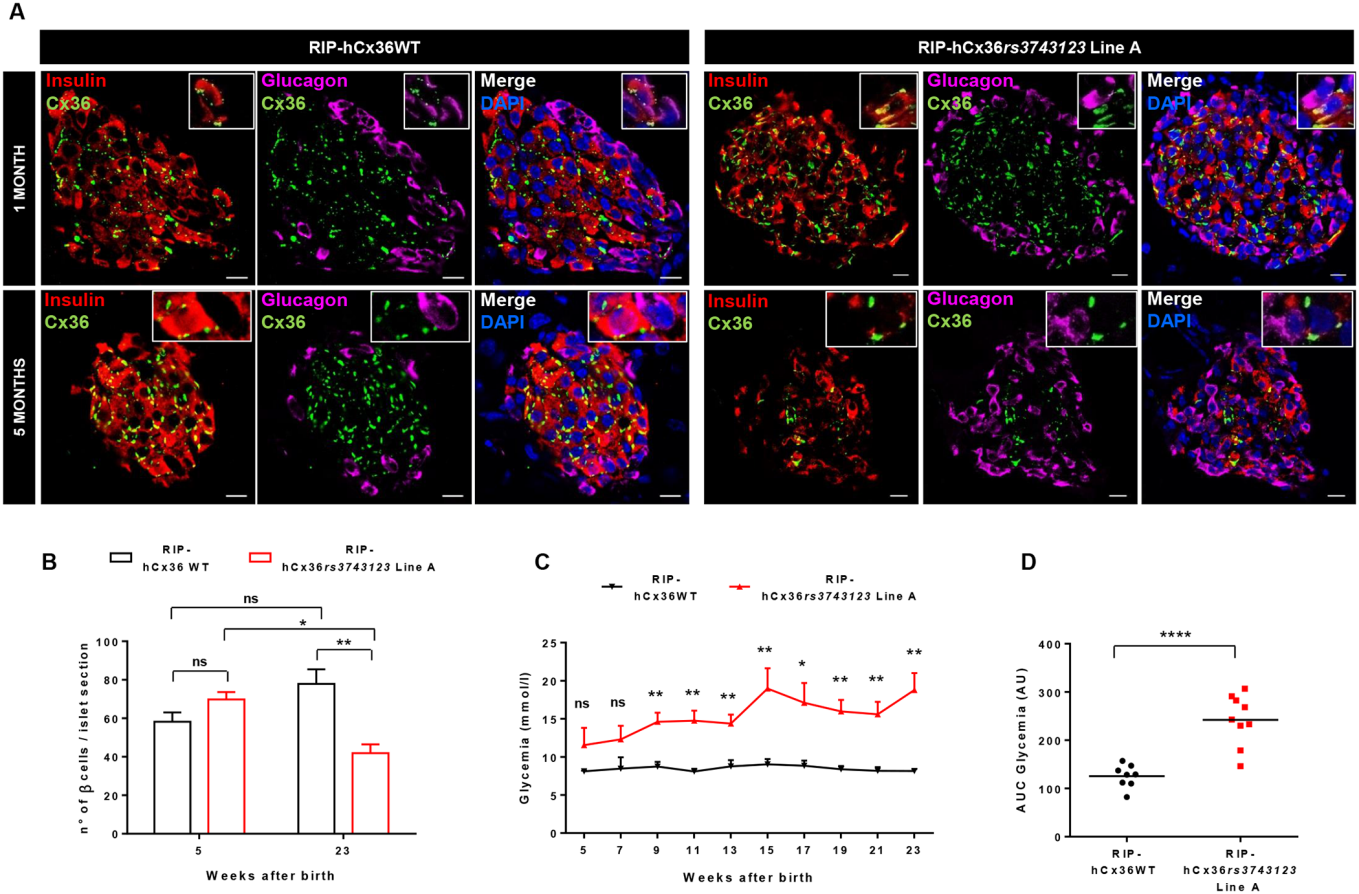
RIP-hCx36SNP*rs3743123* mice display β-cell loss and impaired glucose control over time. **A,** Immunofluorescence images of islets of RIP-hCx36*rs3743123* and RIP-hCx36WT mice. In young mice, islets display a normal morphology with α cells in the periphery and β cells at the center. Aging does not alter the islets of RIP-hCx36WT mice but determines loss of organization of α and β cells in RIP-hCx36*rs3743123* mice. Scale bar 10 μm. **B,** Number of β cells per islet section in islets of RIP-hCx36WT and RIP-hCx36*rs3743123* mice. Compared to 1 month old littermates, 5 month old RIP-hCx36*rs3743123* mice display a decrease of β cells per islet section. Data show means + SEM of islets of 3–9 mice per group. **C,** Glycaemia curve of RIP-hCx36WT and RIP-hCx36*rs3743123* mice. RIP-hCx36 *rs3743123* show increase in blood glucose with aging. D, Area under the entire glycaemia curve. *P ≤ 0.05**P ≤ 0.01***P ≤ 0.001**** P ≤ 0.0001 (Student t-test with Welch’s correction).

Immunofluorescence showed that hCx36 was similarly expressed in the β cells of 1 month-old RIP-hCx36WT and RIP-hCx36*rs3743123* mice (Fig 3) at levels significantly higher than those of native mCx36 of non-transgenic animals (S5B–S5E Fig), consistent with the strong activity of the insulin promoter used to generate the transgenic animals. All 1 month-old mice featured comparable islet architecture, with somatostatin- and glucagon-containing cells located as a mantle at the islet periphery, and surrounding a core of insulin-producing β-cells (S6A and S6B Fig top rows and Fig 3 top row). At this stage, morphometry revealed a comparable number of β-cells in the islets of the two mouse types (Fig 3B).

**Fig 4:**
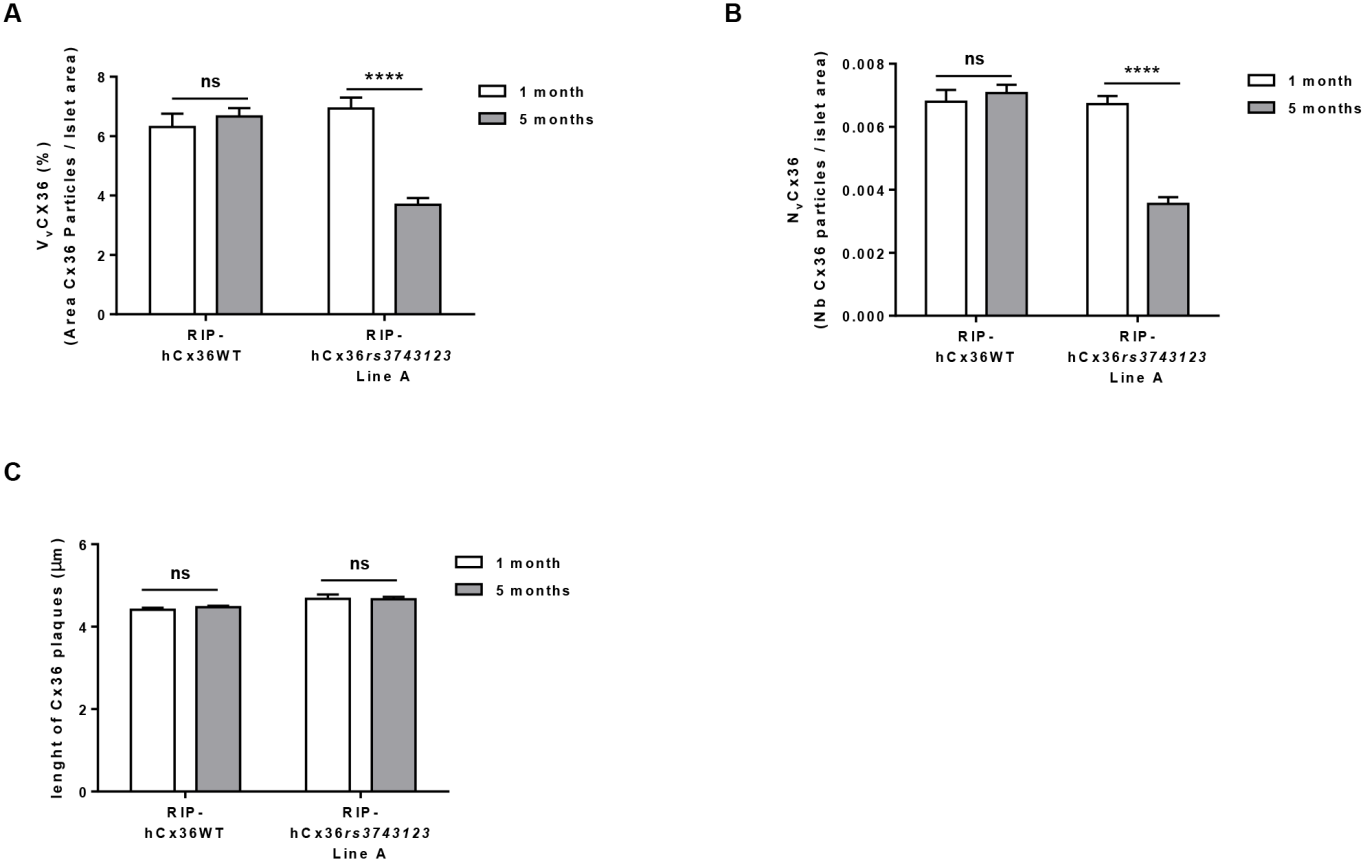
SNP *rs3743123* modify hCx36 expression and its distribution at the beta cell membrane. **A,** The volume density (Vv) of Cx36 decreases postnatally in RipβglohCx36 SNP *rs3743123* mice. **B,** The numeric density (Nv) of Cx36 is reduced with time in RIP-hCx36*rs3743123* mice. **C,** The length of the Cx36 plaques was also decreased postnatally in these mice. Data show means + SEM of islets of three mice per group. *P ≤ 0.05**P ≤ 0.01***P ≤ 0.001**** P ≤ 0.0001 versus 1 month old litters.

Islet structure and proper control of blood glucose levels were not altered with aging in mice expressing the WT form of hCx36 (Fig 3). In contrast, a progressive loss of β-cells (Fig 3A bottom row and Fig 3B), altering islet structure (S6A and S6B Fig bottom rows) and resulting in hyperglycemia (Fig 3C and 3D), was observed with time in one of the two lines (line A) expressing the variant form of hCx36. These alterations, which were observed in male and female mice, and across multiple animal generations, were paralleled by a reduction of hCx36 expression (Fig 4A–4C). Five months after birth, the second line expressing the variant hCx36 (line B), and which was generated independently of line A, also featured a statistically significant reduction in the number of β cells and of immunolabelled hCx36, as compared to mice expressing the WT form of hCx36 (S7A, S7B, S7E–S7H Fig). However, these alterations were substantially milder than those observed in line A. Accordingly, mice of line B remained normoglycemic throughout the entire duration of the experiment (S7C and S7D Fig).

The data show that the overexpression of WT and variant hCx36 did not affect the structure of the endocrine pancreatic islets of young mice, and that the variant connexin later impaired both islet structure and function.

### SNP *rs3743123* reduces *GJD2* expression in human pancreatic islets, but marginally associates to T2D patients

Comparison of the expression of quantitative trait loci (eQTL) in human islets of normoglycaemic and hyperglycaemic donors showed that the expression of hCx36 transcript was significantly decreased (p<0.03) in pancreatic islets of hyperglycemic organ donors, and specifically in those expressing SNP *rs3743123* than in those expressing the WT form of the gene. (S2 Table).

To test whether SNP *rs3743123* associates to T2D, we investigated the CoLaus cohort ([36], S1 Table) for several SNPs which lie in the haplotype block of *rs3743123*, with pair-wise linkage coefficients D’> 0.98 [22]. Five common haplotypes, accounting for 99% of control chromosomes were identified, whose distribution was not significantly different in control and T2D individuals (S3 Table). Comparison of the genotype and allele frequencies of the 4 SNPs we investigated revealed that SNPs *rs651724*, *rs35174018* and *rs34964522* did not associate to T2D in the CoLaus population (S4 Table). In contrast, the synonymous SNP *rs3743123* (S196S) was nominally associated to the disease, at both allele (p = 0.03; OR = 1.27 [1.02–1.82]) and genotype levels, as assessed per a recessive model (p = 0.03; OR = 1.36 [1.02–1.59]), although this association did not reach statistical significance after correction for multiple testing (S4 Table). The data show that SNP *rs3743123* reduces the islet expression of h*GJD2*, but marginally associates to T2D, in a heterogeneous population of patients.

## Discussion

Type 2 diabetes (T2D) refers to a set of pathogenically distinct disorders which are usually identified by a persistent hyperglycemia, often due to a variable loss of the insulin-producing β cells of pancreas, and to a selective insensitivity to glucose of the residual β cells. These alterations are believed to be caused via a complex interplay of multiple genetic, cellular and environmental factors [40, 41]. Previous studies have indicated that Cx36, a protein of the connexin family which forms membrane channels for the electrical and metabolic coupling of β cells [42], contributes to control glucose-stimulated insulin secretion and the resistance of β cells to cytotoxic conditions [18, 42, 43]. Strikingly, the pharmacological, siRNA and genetic interference with the expression and function of Cx36 results in alterations of murine β cells [16, 18, 44], which mimic the major alterations observed in T2D [45].

In humans and rodents, the *GJD2* gene which codes for Cx36 (http://www.genenames.org/genefamily/gj.php) is solely expressed in pancreatic β cells, neurons, and neuron-related endocrines [46]. Previous studies have shown that a specific SNP (*rs3743123*) in the exon 2 of *GJD2*, is associated with juvenile myoclonic epilepsy [21, 22]. Since neurons and β cells share a number of common features [24], in spite of a rather different embryological origin, we investigated whether the same SNP also causes alterations of β-cell function, possibly involved in the development of T2D.

Stable transfection experiments of human cells which lack native connexin expression, demonstrated that the variant cDNA containing SNP *rs3743123* did not alter the expected half-life of the hCx36 transcript, nor prevent the quantitative biosynthesis of the cognate protein, its transport to and insertion within the cell membrane, and its aggregation into *bona fide* gap junction plaques. Still, and in spite of a normal electrophoretic mobility and preservation of the expected immunoreactivity at the C terminus, the hCx36 protein encoded by the variant mRNA formed channels less permeable to two gap junction-permeant tracers, distributed differently within the cell membrane, and aggregated into distinct patterns of gap junction plaques. These observations suggest that the form of hCx36 carrying SNP *rs3743123* is processed somewhat differently than the wild type form. Our computing analysis confirmed that the synonymous SNP *rs3743123* alters the predicted structure of hCx36 mRNA, to an extent which is not induced by other SNPs located in the same exon of *GJD2*. Other synonymous SNPs, have been shown to affect the splicing [47], stability [48], and secondary structure of messenger RNAs [49–51], as well as the levels and function of the cognate proteins [52–55]. For example, differences in the translation rate due long ribosomal pause along mRNAs featuring a SNP can result in major alterations in the co-translational protein folding [53–55], leading to altered protein conformation. Our data now document that, in the case of *GJD2*, the synonymous SNP *rs3743123* did not affect the translation of hCx36, but somehow altered the packing of the protein in the cell membrane, and decreased the permeability of the cognate cell-to-cell channels. Whether the latter change is due to the modified aggregation pattern of connexons and it occurs also *in vivo* remains to be validated. However, based on previous studies, indicating that the experimental modifications of Cx36-dependent coupling induced in cell lines consistently mimicked those found in primary mouse islets exposed to the same conditions [16, 19, 28, 34], the observed decrease in cell-to-cell coupling would be expected to decrease both the secretion and survival of β cells [16, 18, 44].

To validate this possibility, we generated the first lines of transgenic mice expressing in β cells solely the wild type or the variant form of human Cx36. Comparison of these lines showed that they all exhibited normal islet structure and were normoglycemic at birth. However, mice of the two lines expressing the variant form of hCx36 developed a significant reduction in the number of β cells and of Cx36 immuno-labeling within the first five months of life, which persisted in both genders and across many generations and were paralleled by hyperglycemia in one of the lines. Given the independent generation of these two lines, and the hemizyogous genotype of all the mice we used (which was purposely planned to decrease the risk of a positional loss of function), it is very unlikely that the phenotype observed resulted from a random, non-specific, positional effect of the transgene. Rather, the data strengthen the alternative view that this phenotype was specifically due to the expression of the hCx36 variant. However, as sustained hyperglycemia does decrease the β-cell population in many models of diabetic mice [56], and may reduce Cx36 expression [57], the possibility that the altered islet and Cx36 phenotypes we observed were induced by the elevated circulating levels of glucose should also be considered. Two observations do not support this possibility: first, islets of mice expressing *rs3743123*-hCx36 of the A line, which were hyperglycemic, did not show the large increase in α- and δ-cell numbers, which consistently characterizes the abnormal islet architecture in most models of diabetic mice [56]; second, a loss of β-cells was also observed in the islets of line B, which did not featured elevated circulating levels of glucose. Thus, our experiments suggest that the expression of variant hCx36 decreased Cx36 signaling, leading first to a post-natal loss of β cells that, when sufficient, resulted, in turn, in altered homeostasis of blood glucose. This sequence also fits with the finding that the altered islet and Cx36 phenotypes were more intense in mice expressing *rs3743123-*hCx36 of line A, which eventually developed hyperglycemia, than in those of line B, which remained normoglycemic throughout the duration of the experiment. Most likely, this difference can be accounted for by a different level of expression of the hCx36 transgene between the two transgenic lines, which may have been influenced by its site of insertion (due to a different euchromatin environment) and number of copies (due to tandem repeats). Thus, in animals of the A line the lresidual levels of hCx36 may not have suffice to maintain the β-cell mass and, therefore, normal glucose homeostasis, whereas the higher levels of hCx36 expressed in the mice of line B, may have been adequate to sustain both functions. This is in agreement with the previous observations that Cx36 is dispensable for the development and function of pancreatic islets, till more than 50% of the protein is lost [58].

An obvious question is whether our mouse findings apply to humans. In this perspective, it is striking that the islets of cadaveric donors expressing a GJD2 with SNP *rs3743123*, featured a lower expression of Cx36 mRNA than those of individuals expressing the WT form of the gene. Accordingly, our analysis of the CoLaus cohort revealed a nominal, if marginal association of SNP *rs3743123* to T2D patients. However, investigating the DIAGRAM and MAGIC meta-analyses consortia, we failed to detect a significant association of SNP *rs3743123* in other populations of T2D patients (data not shown). Because T2D is a heterogeneous disease, this negative finding does not rule out a relevant contribution of the *GJD2* gene in subpopulations of T2D patients, specifically those who feature the lowest residual insulin secretion in response to glucose stimulation [59], or other phenotypic traits. The validation of this tentative hypothesis awaits the further screening of other patients and transgenic mice populations. At any rate, our data extend to a different and much large human cohort the preliminary findings that hCx36 levels are decreased in some, but not all T2D patients [4], and provide the very first evidence for an *in vivo* pathogenic role of a connexin variant in the function of pancreatic islets.

## Supporting Information

S1 Fig Expression of hCx36 is induced in HeLa cells after transfection of either the WT or the SNP *rs3743123* form of Cx36. **A-B,** Representative western blots of total and membrane protein extracts. hCx36 is induced in several independent clones of HeLa cells stably transfected with either the WT (clones WT#1-2-3) or the SNP *rs3743123* form of hCx36 (clones SNP#1-2-3). **C-D,** Densitometric quantification of Cx36 signal in western blots of total **(C)** and membrane proteins **(D)**. The hCx36 signal was normalized to the actin signal. SNP *rs3743123* does not alter the membrane insertion of Cx36. Data are mean + SD values of three independent experiments. Student's *t* test with Welch's correction. *P ≤ 0.05**P ≤ 0.01***P ≤ 0.001**** P ≤ 0.0001 compared to non-transfected HeLa cells, ^§^ P ≤ 0.05 ^§ §^ P ≤ 0.01 ^§ § §^ P ≤ 0.001 ^§ § § §^ P ≤ 0.0001 compared to Min6 cells.
(PPTX)

S2 Fig Multiple sequence alignment of Cx36 mRNA, the allelic variant 681C>T and the allelic variant 462C>T. The previous versions of the three mRNA are also reported for comparative purposes.
(PPTX)

S3 Fig Predicted structure of the wild type and the *rs3743123* form of hCx36 mRNA. **A,** Wild type hCx36 mRNA. The enlarged section (square) shows the region carrying the 681C. **B**, Folding structure of the *rs3743123* form of Cx36 and magnification of the region carrying the allelic variant 681T.
(PPTX)

S4 Fig Predicted structure of the wild type and the *rs35174018* form of hCx36 mRNA. **A,** mRNA structure of the wild type hCx36 and magnification of the region carrying the 462C. **B**, mRNA structure of hCx36 carrying the allelic variant 462T. Notably, the two structures are conserved. This observation validates the prediction of the altered structure of the Cx36 mRNA 681C>T allelic variant.
(PPTX)

S5 Fig HCx36 overexpression in transgenic animals. **A,** Construct used for generating RIP-hCx36WT and RIP-hCx36*rs3743123* mice. **B-C,** Immunofluorescence images of mouse endogenous hCx36 in islets of wild type and knock out mice. **D-E,** Immunofluorescence images of hCx36 in islets of mice carrying the wild type and the SNP *rs3743123* form of the protein. Scale bar: 10 μm.
(PPTX)

S6 Fig Islets morphology of RIP-hCx36WT and RIP-hCx36*rs3743123* mice. Immunofluorescence images of islets of RIP-hCx36WT (**A**) and RIP-hCx36*rs3743123* mice (**B**) at 1 (top panel) and 5 months (bottom panel) after birth. Somatostatin green, glucagon purple, insulin red. Scale Bar 10 μm.
(PPTX)

S7 Fig Expression of hCx36*rs3743123* causes a mild phenotype in a second, independent mouse line (line B). Immunofluorescence images of islets of RIP-hCx36WT mice, RIP-hCx36*rs3743123* mice of lines A and B, 5 months after birth **(A)** and quantification of the number of β cells per islet section **(B)**. Glycaemia curve **(C)** and area under this curve **(D)** of RIP-hCx36*rs3743123* line B mice. Immunofluorescence images of hCx36 in islets of RIP-hCx36*rs3743123* line B mice 1 and 5 months after birth **(E)**. Quantification of volume density (Vv) **(F)**, numeric density (Nv) **(G)**, and length of hCx36 plaques **(H)** in RIP-hCx36*rs3743123* mice of the B line. Data show means + SEM. *P ≤ 0.05**P ≤ 0.01***P ≤ 0.001**** P ≤ 0.0001.
(PPTX)

S1 Table Characteristics of the T2D and control groups from the CoLaus cohort analysed to establish the distribution of *GJD2* SNPs. (PPTX)

S2 Table Control of *GJD2* transcription in human islets by SNP *rs3743123*. (PPTX)

S3 Table Distribution of *GJD2* haplotypes in CoLaus cohort. (PPTX)

S4 Table Case-control association studies of 4 SNPs in exon 2 of *GJD2* in the CoLaus study. (PPTX)
