# Supplementary material for: A Variant of GJD2, Encoding for Connexin 36, Alters the Function of Insulin Producing β-Cells
Source: PLoS One. 2016 Mar 9;11(3):e0150880. doi: 10.1371/journal.pone.0150880 (PMC4784816; doi:10.1371/journal.pone.0150880)
Supplement: S1 Fig — A-B, Representative western blots of total and membrane protein extracts. hCx36 is induced in several independent clones of HeLa cells stably transfected with either the WT (clones WT#1-2-3) or the SNP rs3743123 form of hCx36 (clones SNP#1-2-3). C-D, Densitometric quantification of Cx36 signal in western blots of total (C) and membrane proteins (D). The hCx36 signal was normalized to the actin signal. SNP rs3743123 does not alter the membrane insertion of Cx36. Data are mean + SD values of three independent experiments. Student's t test with Welch's correction. *P ≤ 0.05**P ≤ 0.01***P ≤ 0.001**** P ≤ 0.0001 compared to non-transfected HeLa cells, § P ≤ 0.05 § § P ≤ 0.01 § § § P ≤ 0.001 § § § § P ≤ 0.0001 compared to Min6 cells. (PPTX) [file pone.0150880.s001.pptx]

## Slide 1
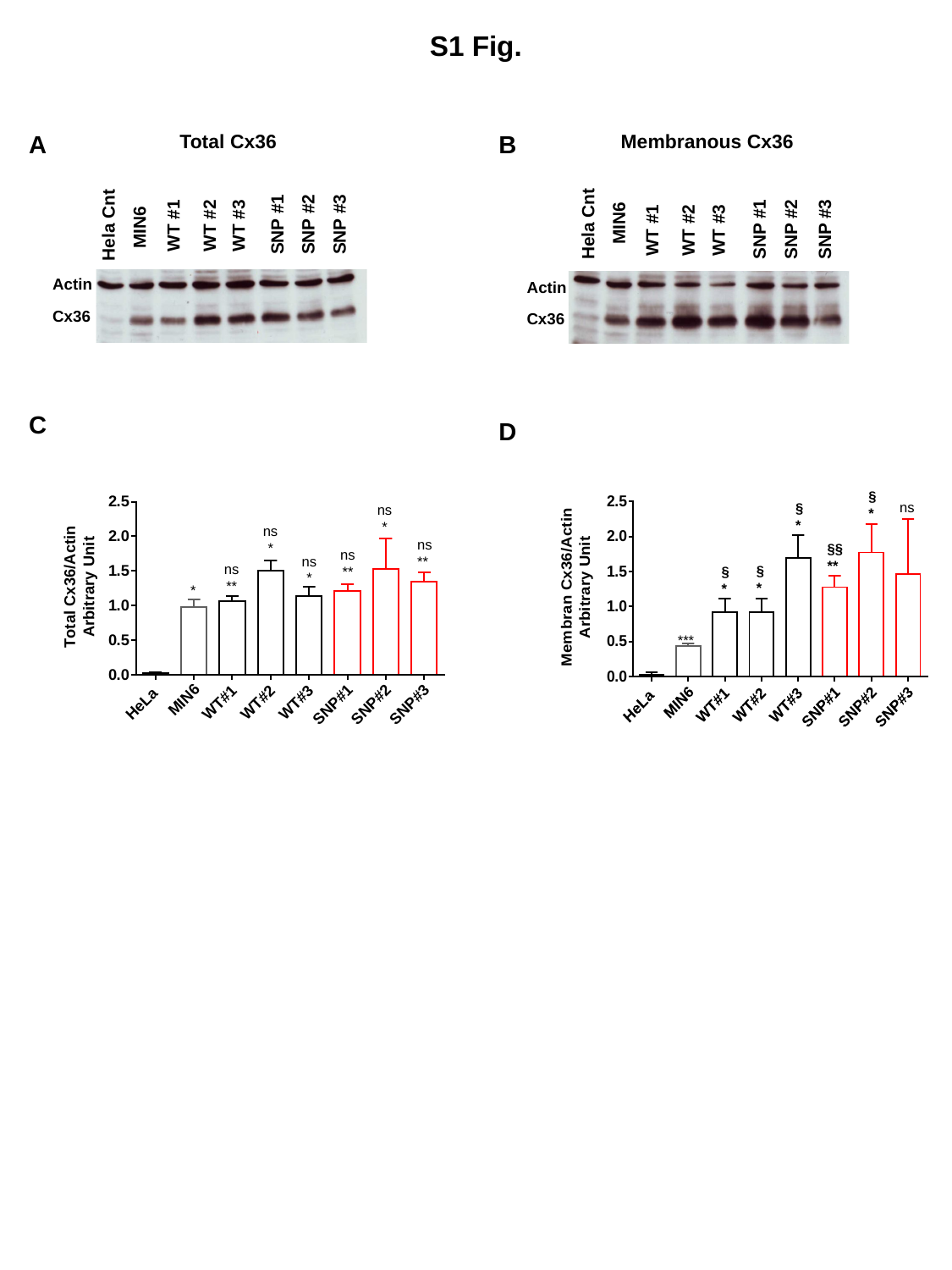

S1 Fig.
A
Total Cx36
B
Membranous Cx36
MIN6
Hela Cnt
SNP #1
SNP #2
SNP #3
Hela Cnt
WT #1
WT #2
WT #3
MIN6
SNP #1
SNP #2
SNP #3
WT #1
WT #2
WT #3
Actin
Actin
Cx36
Cx36
C
D
§
*
ns
§
*
ns
*
ns
*
ns
**
§§
**
ns
**
ns
*
ns
**
§
*
§
*
*
***
