# Supplementary figures and images for: A Variant of GJD2, Encoding for Connexin 36, Alters the Function of Insulin Producing β-Cells
Source: PLoS One. 2016 Mar 9;11(3):e0150880. doi: 10.1371/journal.pone.0150880 (PMC4784816; doi:10.1371/journal.pone.0150880)

## Slide 1
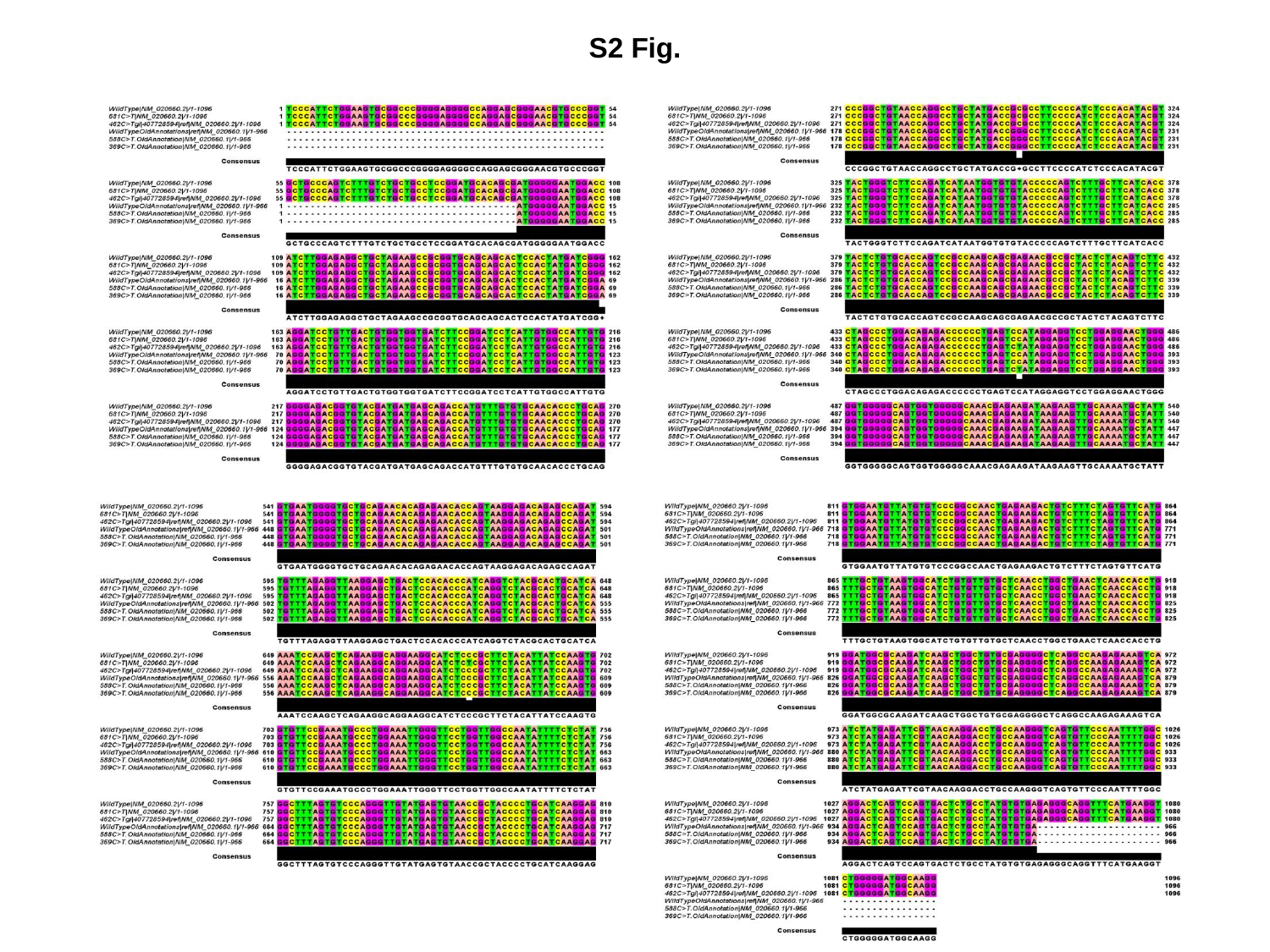

S2 Fig.

Supplement: S2 Fig — The previous versions of the three mRNA are also reported for comparative purposes. (PPTX) [file pone.0150880.s002.pptx]

## Slide 1
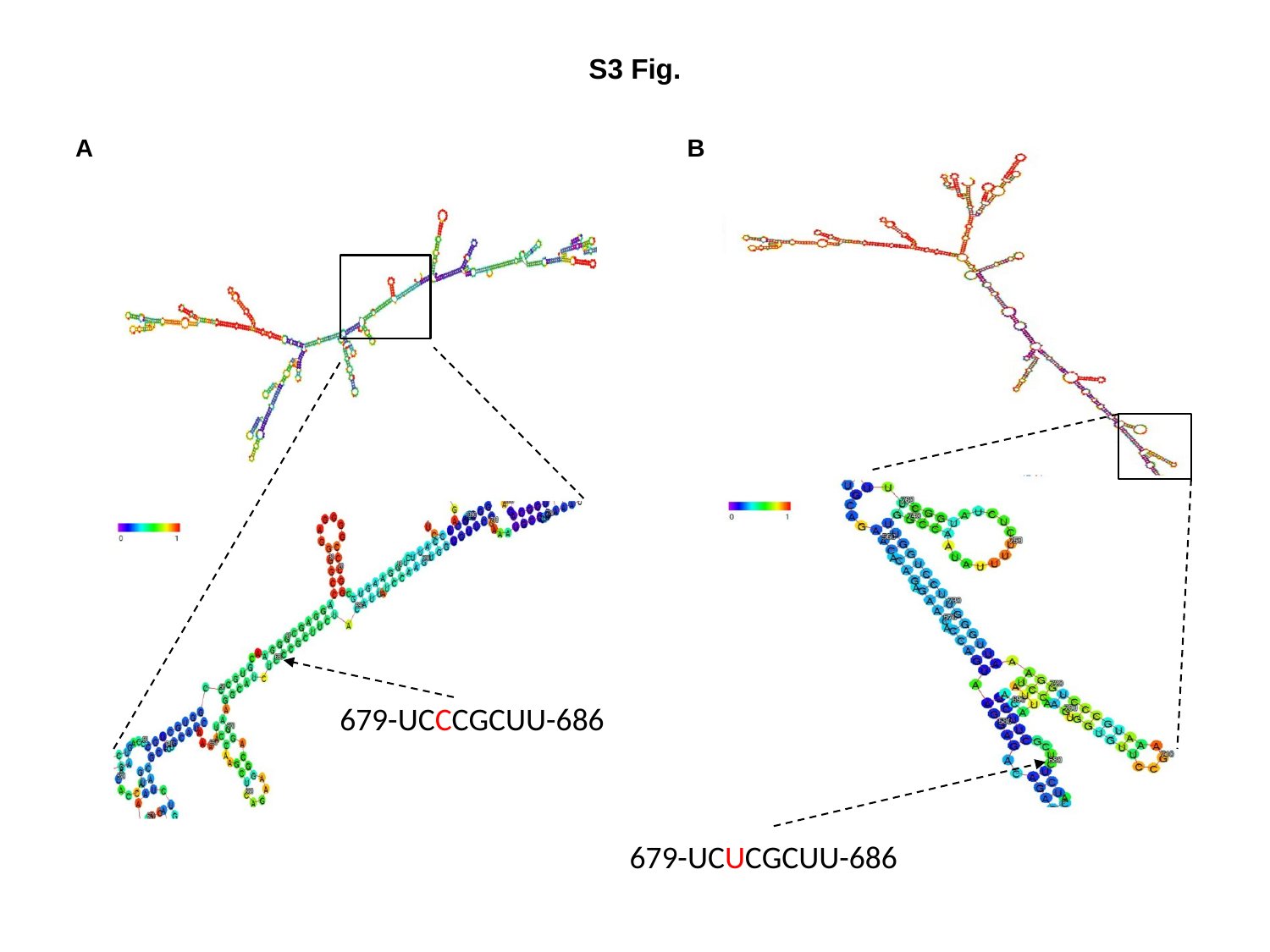

S3 Fig.
A
679-UCUCGCUU-686
B
679-UCCCGCUU-686

Supplement: S3 Fig — A, Wild type hCx36 mRNA. The enlarged section (square) shows the region carrying the 681C. B, Folding structure of the rs3743123 form of Cx36 and magnification of the region carrying the allelic variant 681T. (PPTX) [file pone.0150880.s003.pptx]

## Slide 1
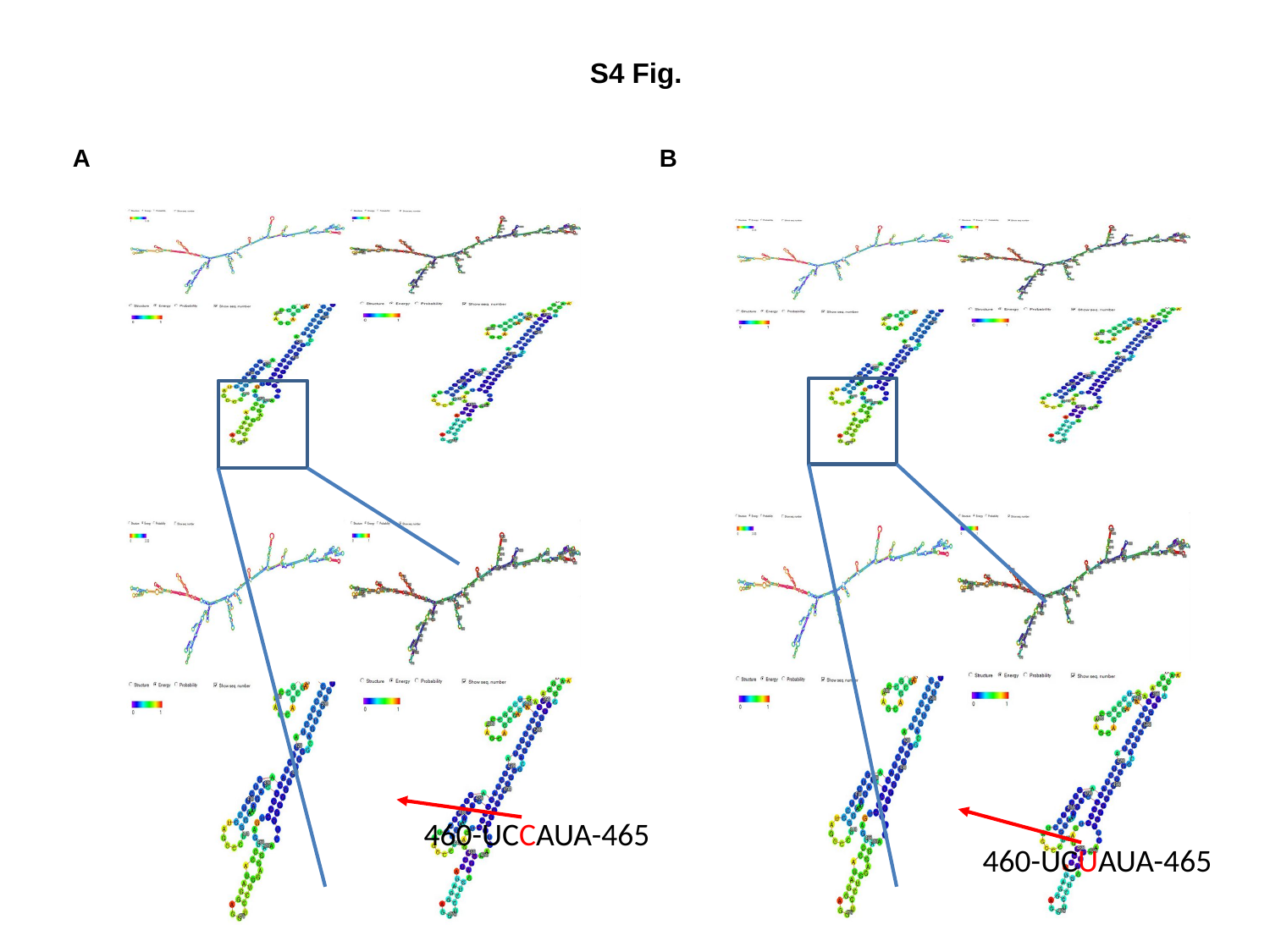

S4 Fig.
A
B
460-UCUAUA-465
460-UCCAUA-465

Supplement: S4 Fig — A, mRNA structure of the wild type hCx36 and magnification of the region carrying the 462C. B, mRNA structure of hCx36 carrying the allelic variant 462T. Notably, the two structures are conserved. This observation validates the prediction of the altered structure of the Cx36 mRNA 681C>T allelic variant. (PPTX) [file pone.0150880.s004.pptx]
