# Supplementary material for: A Variant of GJD2, Encoding for Connexin 36, Alters the Function of Insulin Producing β-Cells
Source: PLoS One. 2016 Mar 9;11(3):e0150880. doi: 10.1371/journal.pone.0150880 (PMC4784816; doi:10.1371/journal.pone.0150880)
Supplement: S5 Fig — A, Construct used for generating RIP-hCx36WT and RIP-hCx36rs3743123 mice. B-C, Immunofluorescence images of mouse endogenous hCx36 in islets of wild type and knock out mice. D-E, Immunofluorescence images of hCx36 in islets of mice carrying the wild type and the SNP rs3743123 form of the protein. Scale bar: 10 μm. (PPTX) [file pone.0150880.s005.pptx]

## Slide 1
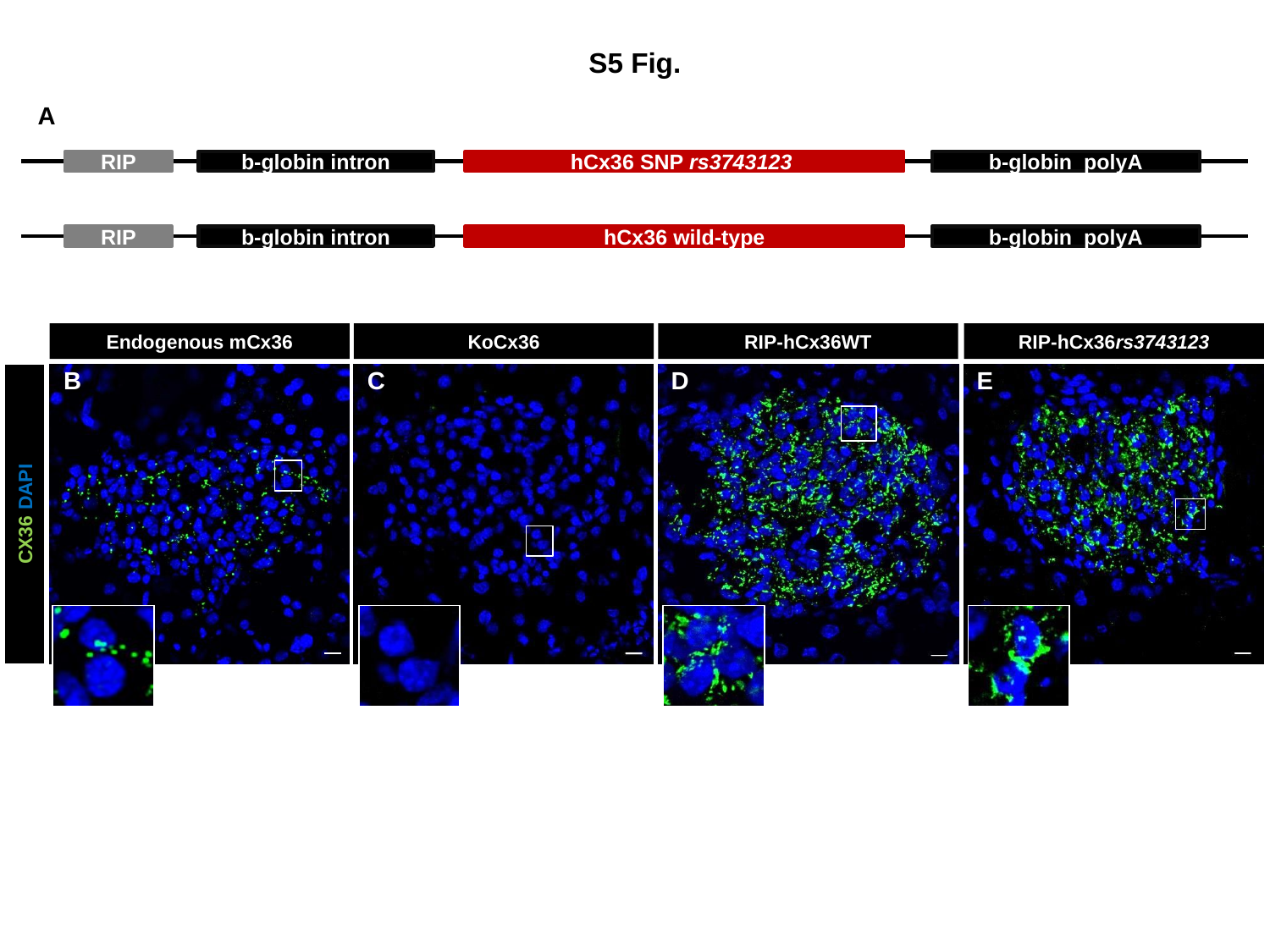

S5 Fig.
A
hCx36 SNP rs3743123
RIP
b-globin intron
b-globin polyA
hCx36 wild-type
RIP
b-globin intron
b-globin polyA
Endogenous mCx36
KoCx36
RIP-hCx36WT
RIP-hCx36rs3743123
B
C
D
E
CX36 DAPI
