# Supplementary material for: A Variant of GJD2, Encoding for Connexin 36, Alters the Function of Insulin Producing β-Cells
Source: PLoS One. 2016 Mar 9;11(3):e0150880. doi: 10.1371/journal.pone.0150880 (PMC4784816; doi:10.1371/journal.pone.0150880)
Supplement: S6 Fig — Immunofluorescence images of islets of RIP-hCx36WT (A) and RIP-hCx36rs3743123 mice (B) at 1 (top panel) and 5 months (bottom panel) after birth. Somatostatin green, glucagon purple, insulin red. Scale Bar 10 μm. (PPTX) [file pone.0150880.s006.pptx]

## Slide 1
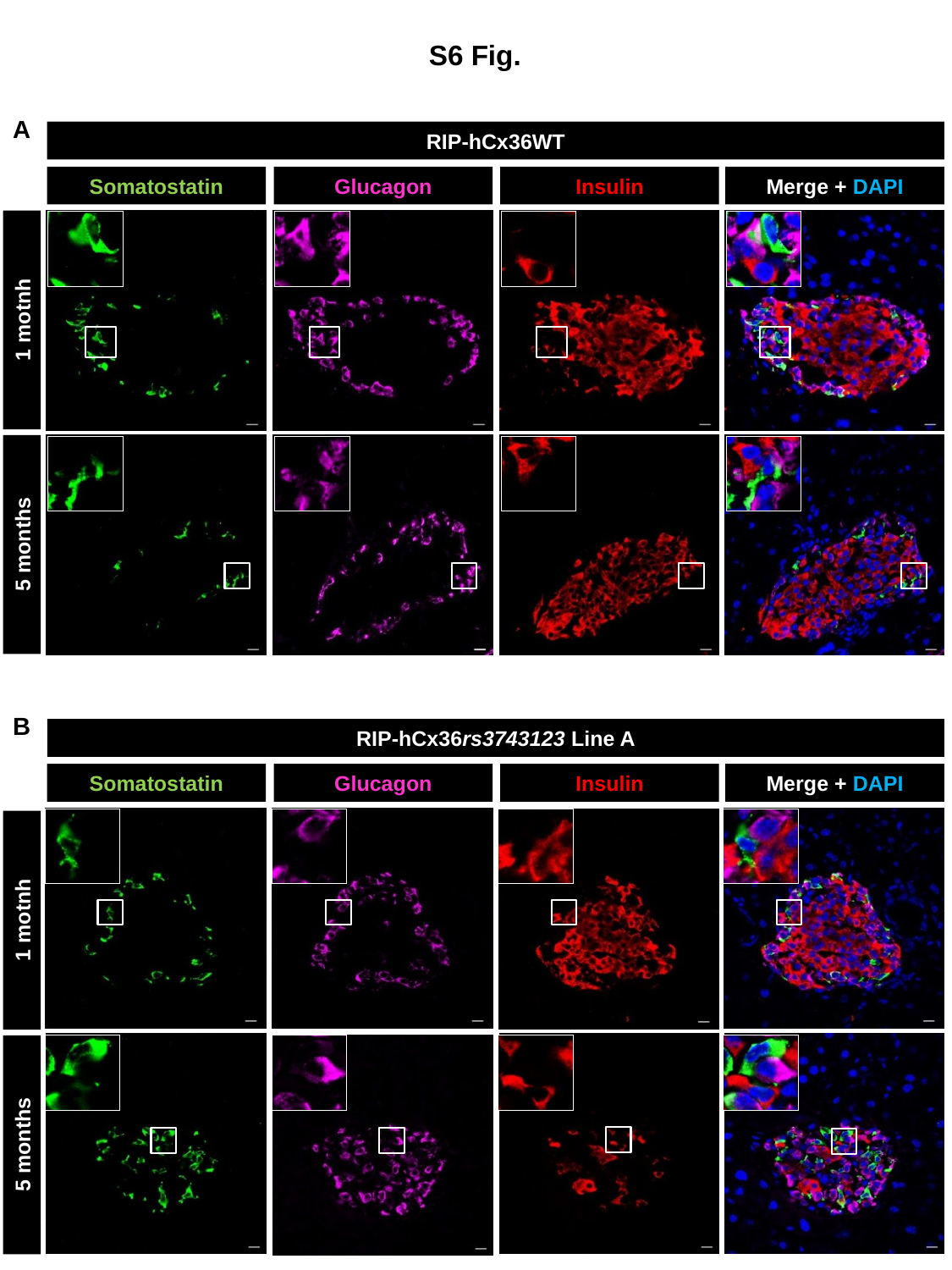

S6 Fig.
A
RIP-hCx36WT
Somatostatin
Glucagon
Insulin
Merge + DAPI
1 motnh
5 months
B
RIP-hCx36rs3743123 Line A
Somatostatin
Glucagon
Insulin
Merge + DAPI
1 motnh
5 months
