# Supplementary material for: A Variant of GJD2, Encoding for Connexin 36, Alters the Function of Insulin Producing β-Cells
Source: PLoS One. 2016 Mar 9;11(3):e0150880. doi: 10.1371/journal.pone.0150880 (PMC4784816; doi:10.1371/journal.pone.0150880)
Supplement: S7 Fig — Immunofluorescence images of islets of RIP-hCx36WT mice, RIP-hCx36rs3743123 mice of lines A and B, 5 months after birth (A) and quantification of the number of β cells per islet section (B). Glycaemia curve (C) and area under this curve (D) of RIP-hCx36rs3743123 line B mice. Immunofluorescence images of hCx36 in islets of RIP-hCx36rs3743123 line B mice 1 and 5 months after birth (E). Quantification of volume density (Vv) (F), numeric density (Nv) (G), and length of hCx36 plaques (H) in RIP-hCx36rs3743123 mice of the B line. Data show means + SEM. *P ≤ 0.05**P ≤ 0.01***P ≤ 0.001**** P ≤ 0.0001. (PPTX) [file pone.0150880.s007.pptx]

## Slide 1
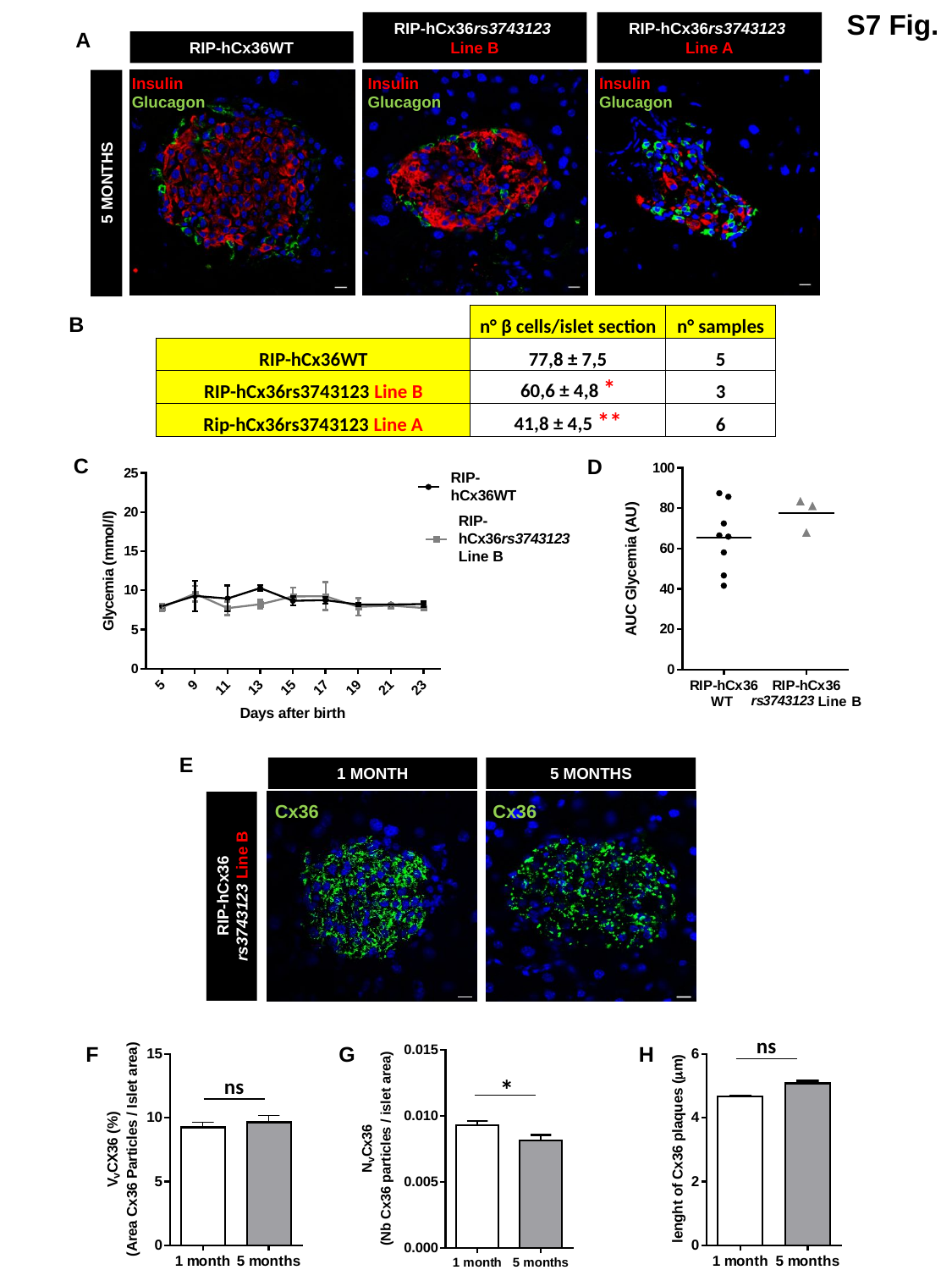

S7 Fig.
RIP-hCx36rs3743123
Line B
RIP-hCx36rs3743123
Line A
RIP-hCx36WT
A
Insulin
Glucagon
Insulin
Glucagon
Insulin
Glucagon
5 MONTHS
B
| | n° β cells/islet section | n° samples |
| --- | --- | --- |
| RIP-hCx36WT | 77,8 ± 7,5 | 5 |
| RIP-hCx36rs3743123 Line B | 60,6 ± 4,8 \* | 3 |
| Rip-hCx36rs3743123 Line A | 41,8 ± 4,5 \*\* | 6 |
C
D
E
1 MONTH
5 MONTHS
RIP-hCx36
rs3743123 Line B
Cx36
Cx36
ns
F
G
H
*
ns
