# Supplementary material for: A Variant of GJD2, Encoding for Connexin 36, Alters the Function of Insulin Producing β-Cells
Source: PLoS One. 2016 Mar 9;11(3):e0150880. doi: 10.1371/journal.pone.0150880 (PMC4784816; doi:10.1371/journal.pone.0150880)
Supplement: S1 Table — (PPTX) [file pone.0150880.s008.pptx]

## Slide 1
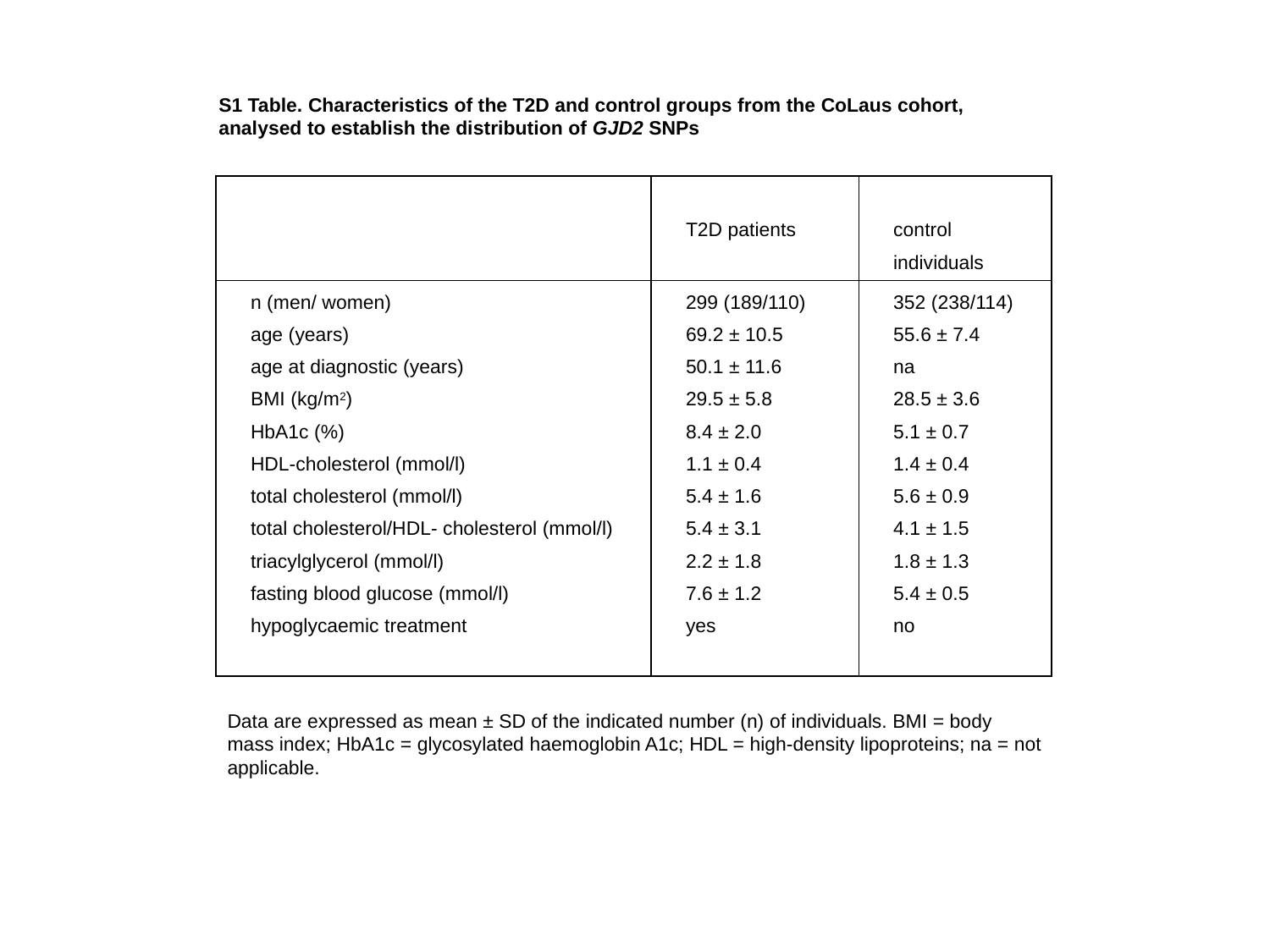

S1 Table. Characteristics of the T2D and control groups from the CoLaus cohort,
analysed to establish the distribution of GJD2 SNPs
| | T2D patients | control individuals |
| --- | --- | --- |
| n (men/ women) age (years) age at diagnostic (years) BMI (kg/m2) HbA1c (%) HDL-cholesterol (mmol/l) total cholesterol (mmol/l) total cholesterol/HDL- cholesterol (mmol/l) triacylglycerol (mmol/l) fasting blood glucose (mmol/l) hypoglycaemic treatment | 299 (189/110) 69.2 ± 10.5 50.1 ± 11.6 29.5 ± 5.8 8.4 ± 2.0 1.1 ± 0.4 5.4 ± 1.6 5.4 ± 3.1 2.2 ± 1.8 7.6 ± 1.2 yes | 352 (238/114) 55.6 ± 7.4 na 28.5 ± 3.6 5.1 ± 0.7 1.4 ± 0.4 5.6 ± 0.9 4.1 ± 1.5 1.8 ± 1.3 5.4 ± 0.5 no |
Data are expressed as mean ± SD of the indicated number (n) of individuals. BMI = body mass index; HbA1c = glycosylated haemoglobin A1c; HDL = high-density lipoproteins; na = not applicable.
