# Supplementary material for: A Variant of GJD2, Encoding for Connexin 36, Alters the Function of Insulin Producing β-Cells
Source: PLoS One. 2016 Mar 9;11(3):e0150880. doi: 10.1371/journal.pone.0150880 (PMC4784816; doi:10.1371/journal.pone.0150880)
Supplement: S2 Table — (PPTX) [file pone.0150880.s009.pptx]

## Slide 1
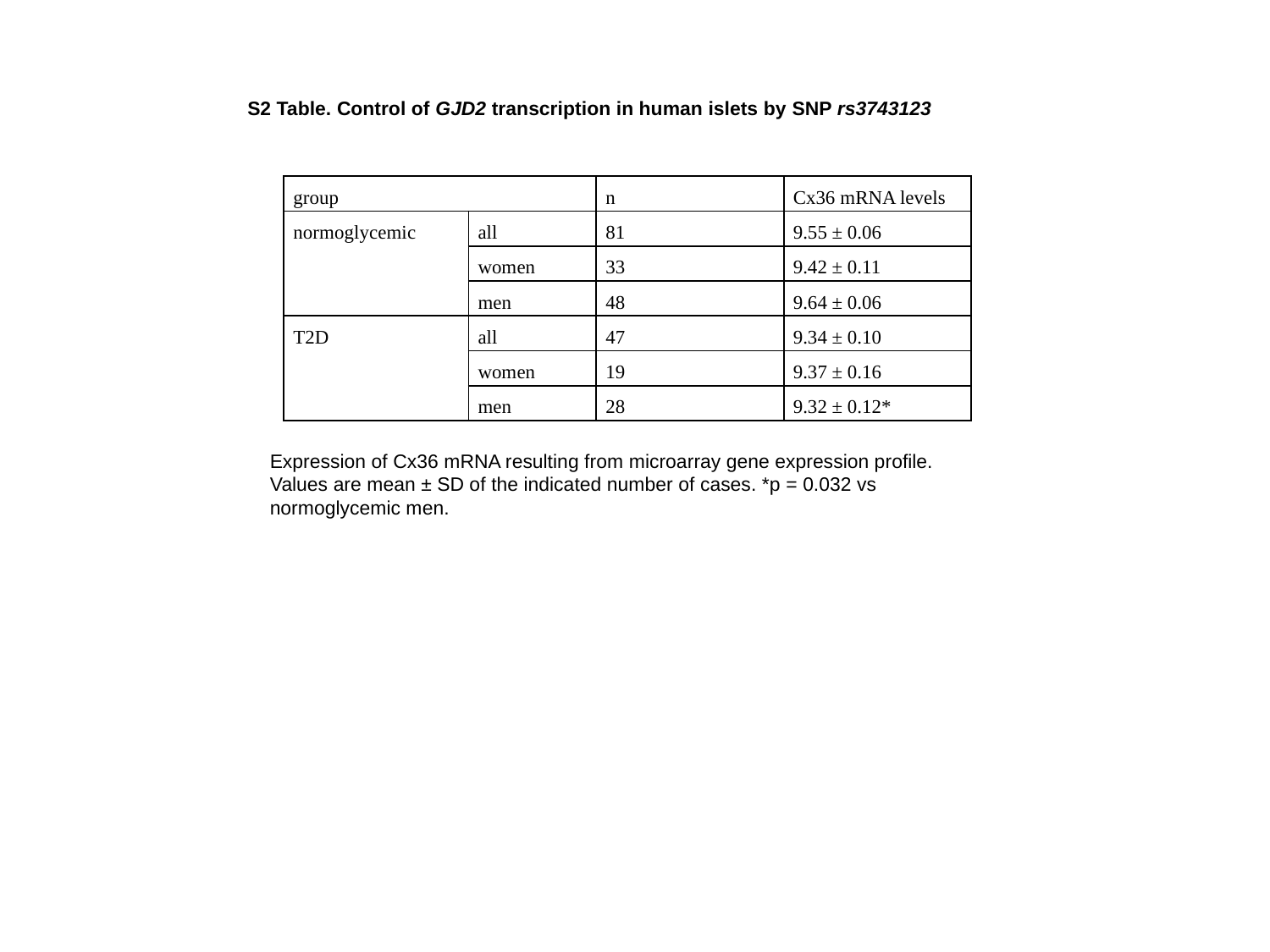

S2 Table. Control of GJD2 transcription in human islets by SNP rs3743123
| group | | n | Cx36 mRNA levels |
| --- | --- | --- | --- |
| normoglycemic | all | 81 | 9.55 ± 0.06 |
| | women | 33 | 9.42 ± 0.11 |
| | men | 48 | 9.64 ± 0.06 |
| T2D | all | 47 | 9.34 ± 0.10 |
| | women | 19 | 9.37 ± 0.16 |
| | men | 28 | 9.32 ± 0.12\* |
Expression of Cx36 mRNA resulting from microarray gene expression profile.
Values are mean ± SD of the indicated number of cases. *p = 0.032 vs normoglycemic men.
