# Supplementary material for: A Variant of GJD2, Encoding for Connexin 36, Alters the Function of Insulin Producing β-Cells
Source: PLoS One. 2016 Mar 9;11(3):e0150880. doi: 10.1371/journal.pone.0150880 (PMC4784816; doi:10.1371/journal.pone.0150880)
Supplement: S3 Table — (PPTX) [file pone.0150880.s010.pptx]

## Slide 1
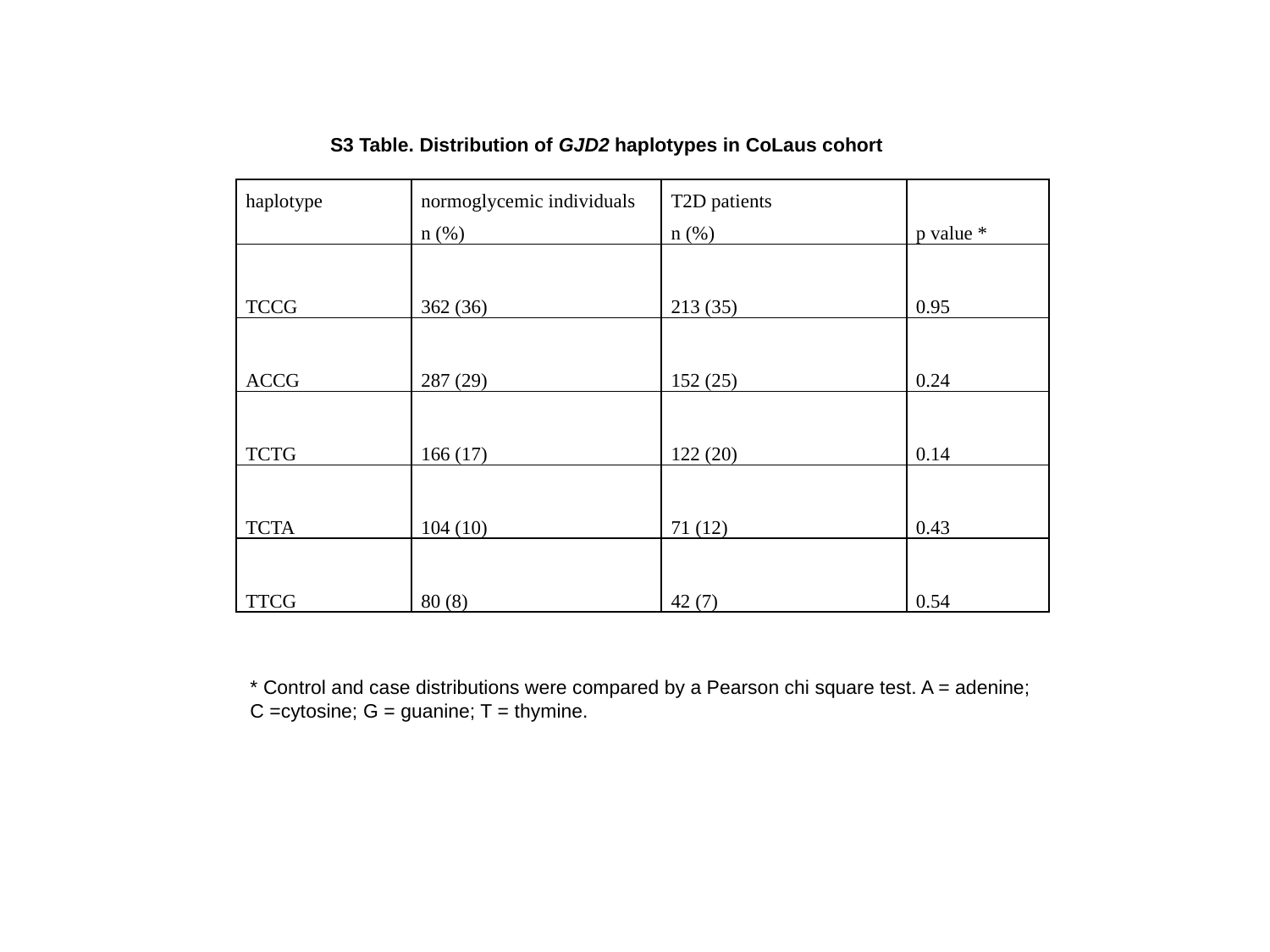

S3 Table. Distribution of GJD2 haplotypes in CoLaus cohort
| haplotype | normoglycemic individuals n (%) | T2D patients n (%) | p value \* |
| --- | --- | --- | --- |
| TCCG | 362 (36) | 213 (35) | 0.95 |
| ACCG | 287 (29) | 152 (25) | 0.24 |
| TCTG | 166 (17) | 122 (20) | 0.14 |
| TCTA | 104 (10) | 71 (12) | 0.43 |
| TTCG | 80 (8) | 42 (7) | 0.54 |
* Control and case distributions were compared by a Pearson chi square test. A = adenine; C =cytosine; G = guanine; T = thymine.
