# Supplementary material for: A Variant of GJD2, Encoding for Connexin 36, Alters the Function of Insulin Producing β-Cells
Source: PLoS One. 2016 Mar 9;11(3):e0150880. doi: 10.1371/journal.pone.0150880 (PMC4784816; doi:10.1371/journal.pone.0150880)
Supplement: S4 Table — (PPTX) [file pone.0150880.s011.pptx]

## Slide 1
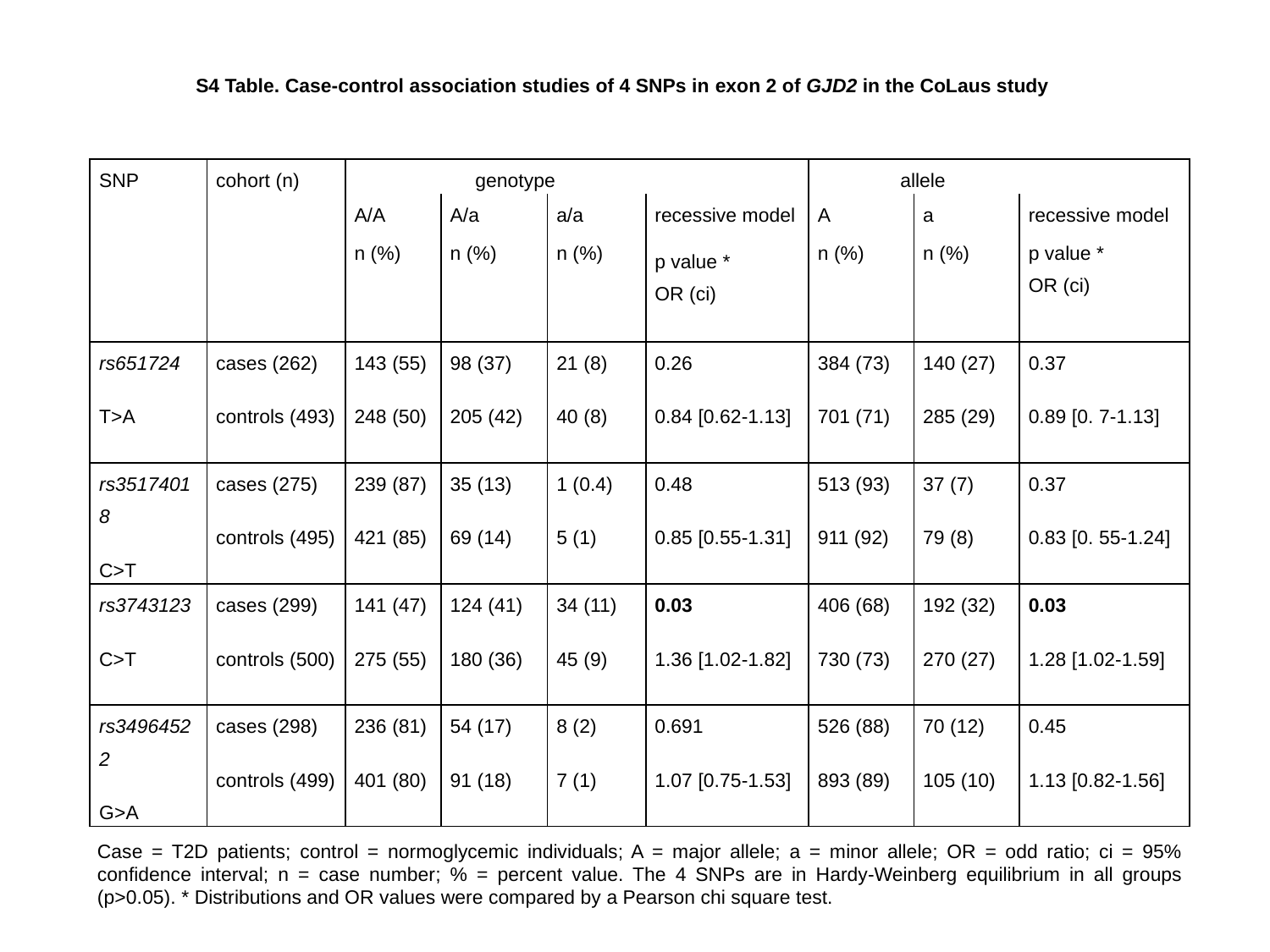

S4 Table. Case-control association studies of 4 SNPs in exon 2 of GJD2 in the CoLaus study
| SNP | cohort (n) | genotype | | | | allele | | |
| --- | --- | --- | --- | --- | --- | --- | --- | --- |
| | | A/A n (%) | A/a n (%) | a/a n (%) | recessive model p value \* OR (ci) | A n (%) | a n (%) | recessive model p value \* OR (ci) |
| rs651724 T>A | cases (262) controls (493) | 143 (55) 248 (50) | 98 (37) 205 (42) | 21 (8) 40 (8) | 0.26 0.84 [0.62-1.13] | 384 (73) 701 (71) | 140 (27) 285 (29) | 0.37 0.89 [0. 7-1.13] |
| rs35174018 C>T | cases (275) controls (495) | 239 (87) 421 (85) | 35 (13) 69 (14) | 1 (0.4) 5 (1) | 0.48 0.85 [0.55-1.31] | 513 (93) 911 (92) | 37 (7) 79 (8) | 0.37 0.83 [0. 55-1.24] |
| rs3743123 C>T | cases (299) controls (500) | 141 (47) 275 (55) | 124 (41) 180 (36) | 34 (11) 45 (9) | 0.03 1.36 [1.02-1.82] | 406 (68) 730 (73) | 192 (32) 270 (27) | 0.03 1.28 [1.02-1.59] |
| rs34964522 G>A | cases (298) controls (499) | 236 (81) 401 (80) | 54 (17) 91 (18) | 8 (2) 7 (1) | 0.691 1.07 [0.75-1.53] | 526 (88) 893 (89) | 70 (12) 105 (10) | 0.45 1.13 [0.82-1.56] |
Case = T2D patients; control = normoglycemic individuals; A = major allele; a = minor allele; OR = odd ratio; ci = 95% confidence interval; n = case number; % = percent value. The 4 SNPs are in Hardy-Weinberg equilibrium in all groups (p>0.05). * Distributions and OR values were compared by a Pearson chi square test.
